# Turbulent Systolic Flow Downstream of a Bioprosthetic Aortic Valve: Velocity Spectra, Wall Shear Stresses, and Turbulent Dissipation Rates

**DOI:** 10.3389/fphys.2020.577188

**Published:** 2020-09-29

**Authors:** Barna Becsek, Leonardo Pietrasanta, Dominik Obrist

**Affiliations:** ARTORG Center for Biomedical Engineering Research, University of Bern, Bern, Switzerland

**Keywords:** bioprosthetic heart valve, leaflet fluttering, thrombocyte activation, wall shear stress, laminar-turbulent transition, fluid-structure interaction, computational fluid dynamics

## Abstract

Every year, a quarter million patients receive prosthetic heart valves in aortic valve replacement therapy. Prosthetic heart valves are known to lead to turbulent blood flow. This turbulent flow field may have adverse effects on blood itself, on the aortic wall and on the valve performance. A detailed characterization of the turbulent flow downstream of a valve could yield better understanding of its effect on shear-induced thrombocyte activation, unphysiological wall shear stresses and hemodynamic valve performance. Therefore, computational simulations of the flow past a bioprosthetic heart valve were performed. The computational results were validated against experimental measurements of the turbulent flow field with tomographic particle image velocimetry. The turbulent flow was analyzed for disturbance amplitudes, dissipation rates and shear stress distributions. It was found that approximately 26% of the hydrodynamic resistance of the valve was due to turbulent dissipation and that this dissipation mainly took place in a region about one valve diameter downstream of the valve orifice. Farther downstream, the turbulent fluctuations became weaker which was also reflected in the turbulent velocity spectra of the flow field. Viscous shear stresses were found to be in the range of the critical level for blood platelet activation. The turbulent flow led to elevated shear stress levels along the wall of the ascending aorta with strongly fluctuating and chaotic wall shear stress patterns. Further, we identified leaflet fluttering at 40 Hz which was connected to repeated shedding of vortex rings that appeared to feed the turbulent flow downstream of the valve.

## 1. Introduction

Aortic valve replacement (AVR) is a common therapy for moderate to severe aortic stenosis (Kheradvar et al., 2015). In AVR, the diseased native aortic valve is replaced by mechanical (MHV) or biological heart valve prostheses (BHV) in an estimated 250'000 annual interventions worldwide (Yoganathan et al., 2004). Heart valve prostheses are known to create turbulent flow in the aortic root (AR) and the ascending aorta (AAo) (Sotiropoulos et al., 2016). This turbulent flow may be connected to blood platelet activation triggering thrombus formation (Quinlan and Dooley, 2007) and to unphysiological wall shear stresses in the AAo possibly leading to endothelial lesions (Davies et al., 1986). Moreover, turbulent dissipation contributes to the pressure drop across the valve (clinically known as *trans-valvular pressure gradient*, TVPG). Flow instabilities at the level of the valve orifice could be related to leaflet fluttering in BHVs (Peacock, 1990) which may contribute to structural valve deterioration (SVD) in BHVs due to wear and fatigue of the leaflet tissue. Therefore, hydrodynamic instabilities and turbulent flow past BHVs are important phenomena which may be directly linked to clinically adverse events, and it may be advisable to design prostheses which lead to less turbulent flow.

Despite the relevance of turbulent flow past heart valve prostheses, our current understanding of this flow phenomenon remains incomplete. Early investigations of turbulence past heart valve prostheses include a study by Yoganathan et al. (1986) who based their findings on the turbulent shear stresses obtained from a laser Doppler anemometer for several MHV and BHV designs. They found that BHVs exhibit regions of high turbulent shear stresses at the edge of the central aortic jet issuing from the valve orifice during systole. Lim et al. (1998) studied three types of MHVs and one BHV made from porcine tissue with particle image velocimetry (PIV) measurements of steady systolic flow. Their investigation was mainly based on the analysis of Reynolds shear stresses. The authors detected the highest Reynolds shear stresses downstream of the studied BHV at constant flow rate. This result was attributed to the small orifice area of this valve which led to a strongly accelerated aortic jet with sharp shear layers.

The review on heart valve fluid mechanics by Yoganathan et al. (2004) emphasizes the role of the central aortic jet in BHVs and its regions of high shear. It provides a range of values for turbulent stresses that are thought to be relevant for blood platelet activation and thrombus formation. The review by Sotiropoulos et al. (2016) critically assesses the role of the Reynolds stresses as they do not contain information on instantaneous stresses acting on blood cells or platelets. They refer to the study by Ge et al. (2008) who investigated Reynolds and viscous stresses in the wake of a bileaflet MHV and who argued that Reynolds stresses should be used with caution when assessing blood cell damage since they only provide a statistical description on inertial phenomena and generally overestimate the stresses experienced by blood cells. They further emphasized that instantaneous viscous stresses are of greater interest for quantifying regions of excessive stresses on blood particles. The viscous stress levels in the study of Ge et al. (2008) were found to be large enough for platelet activation, while they did not have the magnitude for causing red blood cell damage or hemolysis. A similar finding was reported by Quinlan and Dooley (2007) who based their study on measurements of the flow field in the wake of three different bileaflet MHVs by Liu et al. (2000). Fluctuation values were obtained from phase-averaged quantities. It was shown that for different phases of the heart pulse, the inertial subrange for turbulent momentum transport agreed roughly with Kolmogorov's −5/3–power law (Pope, 2000, p. 230). Quinlan and Dooley (2007) further developed a mathematical model for the estimation of shear stress induced on blood cells based on a spherical cell model. They showed that in laminar flow, the induced blood cell stress is approximately equal to the bulk shear stress of the flow. In turbulent flow, they argue, that the stress experienced by blood particles is approximately one order of magnitude less than the Reynolds shear stresses. They concluded that the Reynolds stresses themselves cannot be used as a sole indicator for cell loading. Rather, the spectral energy distribution of the flow field should be studied and compared to the size of blood particles to identify relevant scales and their fluctuation magnitude for flow-induced blood trauma.

**Figure 1:**
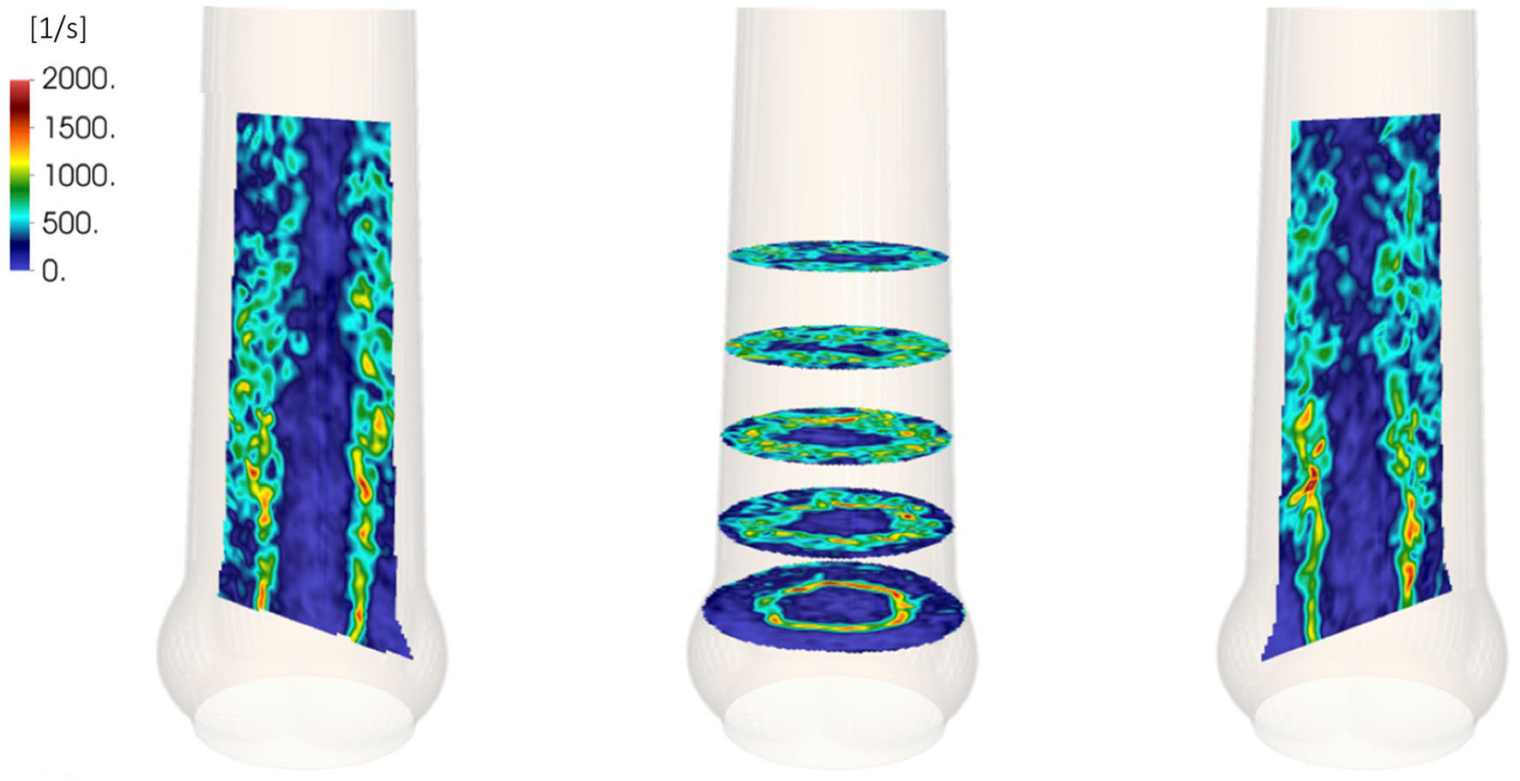
Shear rates in the systolic flow behind a BHV. Adapted from experimental results by Hasler and Obrist (2018).

Among others, Morbiducci et al. (2009), Alemu et al. (2010), and Min Yun et al. (2014) used computational models of bileaflet MHV to study shear-induced platelet activation. The computational study by Hedayat et al. (2017) compared platelet activation in the turbulent flow fields past bileaflet MHV with a model of a BHV and found that the activation potential is higher for MHV. Hedayat and Borazjani (2019) showed that small vortical structures in systolic flow of a bileaflet MHV contribute significantly to their thrombogenic potential. Experimental investigations by Hasler et al. (2016) and Hasler and Obrist (2018) presented three-dimensional PIV data of the flow past a BHV. The measured fields showed turbulent flow with instantaneous shear strain rates beyond 2, 000 s^−1^ (Figure 1). Given that the limited resolution of these PIV measurements probably led to an underestimation of the strain rates, these results indicate that also the turbulent flow behind BHVs may lead to platelet activation.

Despite the intriguing results from Figure 1, the experimental data by Hasler and Obrist (2018) does not allow for a full characterization of the turbulent flow field in the systolic flow downstream of the BHV. The limited spatial resolution of the measurement effectively acts as a low-pass filter which prevents an accurate calculation of turbulent viscous dissipation and wall shear stresses, and the phase-averaged nature of the data renders a temporal analysis of flow phenomena difficult.

Therefore, the present study aims to provide more detailed data on the turbulent systolic flow using a computational model. The results include the statistical characterization of the turbulent flow field during peak systole and a quantification of viscous shear stresses in the bulk flow and of wall shear stresses along the AAo wall. It is shown that approximately 26% of the transvalvular pressure gradient are due to turbulent dissipation. Viscous shear stresses in the turbulent flow can be as high as 40 Pa and turbulent wall shear stresses along the AAo wall are found reach values beyond 12 Pa. Velocity spectra, that were extracted at different locations in the turbulent flow field, exhibit an inertial subrange which adheres to Kolmogorov's −5/3–power law. Furthermore, a vortex shedding phenomenon leading to leaflet fluttering is described and quantified. It is not the aim of this study to directly assess the thrombogenic potential of a specific valve type or to predict other adverse events.

The results of the present study were obtained with a numerical solver for fluid-structure interaction (Nestola et al., 2019) which comprises a high-order flow solver for the simulation of transitional and turbulent flow. The computational model included a BHV and an anatomically correct model of an aortic root including three sinus portions (without coronary ostia). The flow rate was smoothly ramped up until a quasi-steady systolic flow was obtained. This flow state was maintained for 0.1 s to allow for a temporal statistical assessment of the turbulent flow field.

## 2. Methods

### 2.1. Governing Equations

For this study we considered blood as a Newtonian fluid with density ρ_*f*_ and dynamic viscosity μ_*f*_ whose velocity field ***v***_*f*_ = (*v*_*f, x*_, *v*_*f, y*_, *v*_*f, z*_) and pressure *p*_*f*_ was governed by the Navier–Stokes equations for incompressible flow,

(1a) $$\begin{array}{l} \rho_{f}\frac{\partial v_{f}}{\partial t}+\rho_{f}\left(v_{f}\cdot \nabla \right)v_{f}+\nabla p_{f}-\mu_{f}\Delta v_{f}=0 \end{array}$$

(1b) $$\begin{array}{l} \nabla \cdot v_{f}=0 \end{array}$$

The solid structures (BHV, aortic root) satisfied the elastodynamics equations

(1c) $$\begin{array}{l} {\hat{\rho }}_{s}\frac{\partial^{2}{\hat{u}}_{s}}{\partial t^{2}}-\hat{\nabla }\cdot \hat{P}=0 \end{array}$$

where ${\hat{u}}_{s}$ is the displacement field, ${\hat{\rho }}_{s}$ the density of the solid and $\hat{P}$ the Piola–Kirchhoff stress tensor. Boundary conditions

(1d) $$\begin{array}{l} {\hat{u}}_{s}=0 \end{array}$$

were applied at selected boundary points to hold the structures in place within the fluid domain. The symbol $\hat{\cdot }$ indicates values in the reference configuration.

The interaction between solid and fluid was described by interface conditions which were satisfied on the surface of the structures with unit normal vector ***n***,

(1e) $$\begin{array}{l} v_{f}=\frac{u_{s}}{\partial t} \end{array}$$

(1f) $$\begin{array}{l} {\hat{J}}^{-1}\hat{P}{\hat{F}}^{T}n=\sigma_{f}n \end{array}$$

where $\hat{F}=\hat{\nabla }{\hat{u}}_{s}+I$ denotes the deformation gradient plus the identity matrix ***I***, $\hat{J}=\det\hat{F}$ is its associated determinant and **σ**_*f*_ is the Cauchy stress of the fluid. More details on the governing equations of the fluid-structure interaction problem are given in Nestola et al. (2019).

Different constitutive laws were employed to simulate the structural response. The valve ring and aortic root were modeled as linearly elastic. Following the approach of Auricchio et al. (2014), the valve leaflets were modeled using the anisotropic fiber-reinforced Holzapfel–Gasser–Ogden material with two fiber families (Holzapfel et al., 2000). Its Piola–Kirchhoff stress tensor $\hat{P}\left({\hat{u}}_{s}\right)=\partial \text{Ψ}/\partial \hat{F}$ was derived from the scalar strain energy function

(2) $$\begin{array}{l} {\text{Ψ}}_{\text{HGO}}=\frac{\mu_{s}}{2}\left({\bar{I}}_{1}-3\right)+\frac{k_{11}}{2k_{21}}\left(\text{exp}\left[k_{21}{\left({\bar{I}}_{4,1}-1\right)}^{2}\right]-1\right)\\ \;+\frac{k_{12}}{2k_{22}}\left(\text{exp}\left[k_{22}{\left({\bar{I}}_{4,2}-1\right)}^{2}\right]-1\right) \end{array}$$

with constitutive parameters μ_*s*_ for the bulk material mechanics and *k*_*ij*_ for the two fiber families *j* = 1, 2. *Ī*_1_, *Ī*_4, 1_, and *Ī*_4, 2_ denote the modified invariants

(3) $$\begin{array}{l} {\bar{I}}_{1}={\hat{J}}^{-2/3}\text{tr}\left(\hat{C}\right) \end{array}$$

(4) $$\begin{array}{l} {\bar{I}}_{4,1}={\hat{J}}^{-2/3}{\hat{g}}_{0,1}\cdot \hat{C}{\hat{g}}_{0,1} \end{array}$$

(5) $$\begin{array}{l} {\bar{I}}_{4,2}={\hat{J}}^{-2/3}{\hat{g}}_{0,2}\cdot \hat{C}{\hat{g}}_{0,2} \end{array}$$

The fiber orientations were defined by the unit vectors ${\hat{g}}_{0,1}$ and ${\hat{g}}_{0,2}$, while $\hat{C}$ denoted the right Cauchy–Green strain tensor. Incompressibility was controlled by adding a volumetric energy term

(6) $$\begin{array}{l} {\text{Ψ}}_{V}\left(\hat{J}\right)=\kappa /2{\left(\hat{J}-1\right)}^{2} \end{array}$$

with the penalty coefficient κ to the strain energy function.

### 2.2. Model Configuration

We studied the systolic flow through a BHV of nominal size 21 mm which was inserted in a model of an aortic root (AR). The fluid density and viscosity were $\rho_{f}=1,060\text{ kg}/{\text{m}}^{3}$ and μ_*f*_ = 0.006 Pa s which is higher than the typical viscosity of blood (0.003…0.004 Pa s). This viscosity was chosen to reduce the computational cost. The effect of this choice on the results will be critically discussed below. Apart from an overestimation of the stress levels for blood, it will be shown that the overall structure of the flow field is not affected by this choice.

The model was configured to yield an average TVPG (difference between aortic and left ventricular pressure) of 8 mmHg. The flow rate was approximately 14.5l/ min which is a typical systolic flow rate for a mean cardiac output of 4 to 5l/ min (Betts, 2013; Jahren et al., 2015).

#### 2.2.1. Valve Leaflets

**Figure 2:**
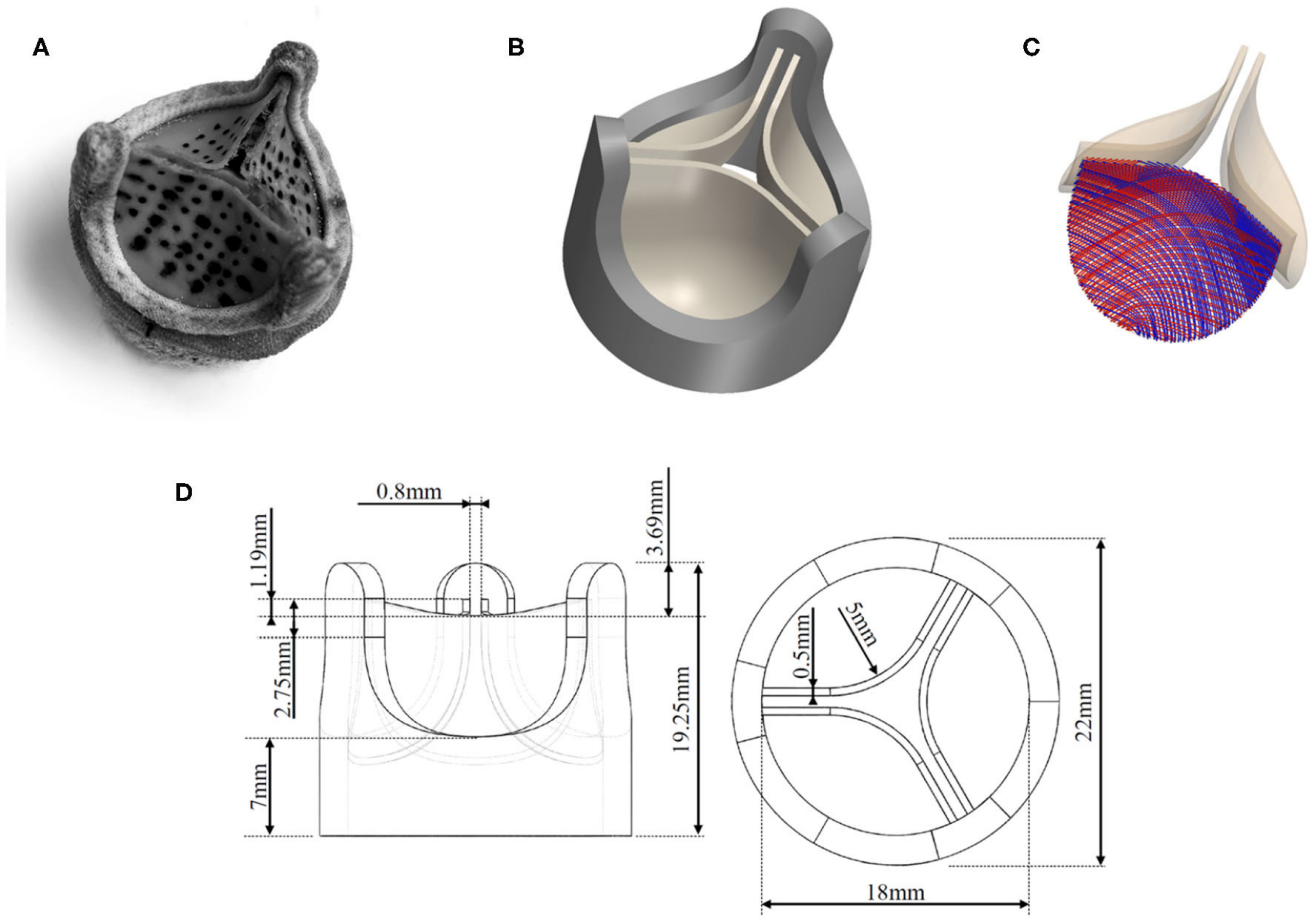
Tri-leaflet bioprosthetic valve model: **(A)** photo of *Edwards Intuity Elite 21* valve by Edwards Lifescience, Irvine, CA, USA, **(B)** CAD model of the valve, **(C)** fiber directions in the leaflet tissue, **(D)** dimensions of the valve model.

The geometry of the valve model was based on a commercial BHV (*Edwards Intuity Elite 21* by Edwards Lifescience, Irvine, CA, USA) valve which was also used in the reference experiments (Figure 2A). Figure 2B shows the corresponding CAD drawing obtained from manually measuring the dimensions of the valve. The dimensions used for the CAD model are presented in Figure 2D. It was assumed that this configuration corresponds to the stress-free reference configuration of the leaflets (Kamensky et al., 2015). The material parameters μ_*s*_ = 20.1 kPa, *k*_11_ = *k*_12_ = 54.62 kPa, and *k*_21_ = *k*_22_ = 30.86 for the Holzapfel–Gasser–Ogden material in Equation (2) were chosen as in Auricchio et al. (2014). Accordingly, the fiber orientation with respect to the horizontal component of the orthogonal basis was set to β = 30° which results in a relative orientation between the two fiber families of 2β = 60° (Figure 2C). Incompressibility of this part of the geometry was achieved through the penalization in Equation (6) with κ = 3·10^4^. The density of the material was set to a value of $\rho_{s}=1,100\text{ kg}/{\text{m}}^{3}$.

#### 2.2.2. Valve Ring

The valve ring (dark gray structure in Figure 2B) comprised all structural elements of the BHV except for the valve leaflets. In the real BHV, the valve ring is made from a thin nitinol wireframe, which shapes the valve posts. The remaining voids in the geometry are then mostly composed from polymer material and covered with textile material. This complex composite structure was modeled as a cylindrical solid ring with three valve posts (dimensions were obtained manually with a caliper and are given in Figure 2D) using a single linearly elastic material. As the mechanical properties for the valve ring were not known, they were estimated and adjusted *ad hoc* to a shear-modulus of μ_*s*_ = 0.6 MPa, a bulk modulus of κ_*s*_ = 3 MPa and a density of $\rho_{s}=1,500\text{ kg}/{\text{m}}^{3}$.

#### 2.2.3. Aortic Root (AR)

**Figure 3:**
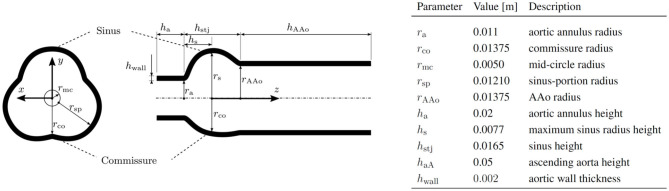
Geometry of the parameterized AR.

**Figure 4:**
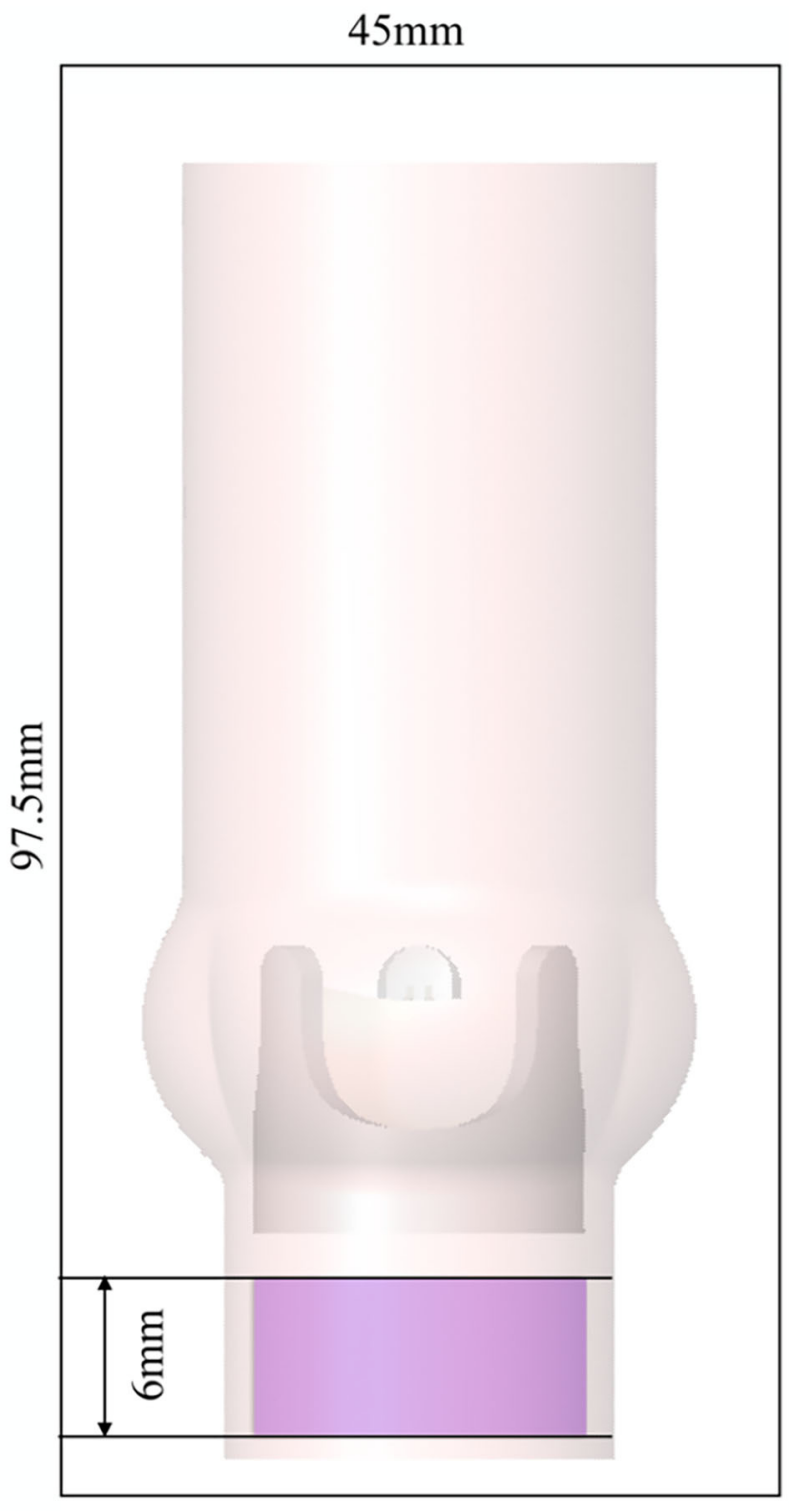
AR and valve model immersed in the fluid domain of size 45 × 45 × 97.5 mm with periodic boundary conditions in all spatial directions. The magenta shaded area indicates where a forcing term was applied.

The geometry for the AR, including the sinus portions and part of the AAo, was a parametrized geometry which was assembled from data by Reul et al. (1990) and Haj-Ali et al. (2012). A silicone phantom based on the same parametrized model has been previously used by Hasler and Obrist (2018) in tomographic PIV measurements (geometry M in that study). Figure 3 shows the AR geometry in a cross-section through the *xy*-plane and a cross-section through the *xz*-plane together with the values of the individual radii and heights of the sections. The complete setup used for the simulations is shown in Figure 4, which also includes the dimensions of the computational domain for the fluid.

The coordinate system (*x, y, z*) originates at a point on the central axis of the AR at a height corresponding to the maximum sinus radius *r*_*s*_. The sinotubular junction (STJ) is an important landmark located at *z*_stj_ = 0.0088 m and marks the point of transition from the bulbous sinus portion to the straight AAo.

The AR was modeled as a linearly elastic material with a shear-modulus μ_*s*_ = 0.3 MPa, a bulk modulus of κ_*s*_ = 3 MPa and a density of $\rho_{s}=1,100\text{ kg}/{\text{m}}^{3}$. We chose a linear elastic material description because (a) the resulting strains in the AR remained small and (b) the numerical results were compared to experimental results with an aortic root phantom manufactured from silicone (Hasler and Obrist, 2018). The chosen values of μ_*s*_ and κ_*s*_ therefore yield a material that corresponds to a volume ratio of 1:5 between curing agent and polymer according to Armani et al. (1999) if the Poisson ratio is chosen to be ν_*s*_ = 0.45.

### 2.3. Numerical Method

The governing Equation (1) were solved with a dedicated solver for fluid-structure interaction problems Nestola et al. (2019) which is based on the immersed boundary method (Peskin, 2002). To this end, the valve and the AR were immersed in a computational fluid domain with dimensions 45 × 45 × 97.5 mm (Figure 4). The flow was discretized with a 6^th^-order finite difference scheme on a rectilinear grid with 129 × 129 × 193 grid points. Grid stretching yielded increased resolution in the center of the fluid domain with a mesh width below 0.25 mm.

The structure was discretized with a finite-element method using approximately 98'000 ℙ1 tetrahedral elements (73'163 for the AR; 18'204 for the valve ring; 6'523 for the valve leaflets). Data was transferred between fluid and structural meshes using a variational formulation of transfer operators for arbitrary meshes (Krause and Zulian, 2016). The reader is referred to Nestola et al. (2019) for details on the numerical implementation, convergence and code validation.

The simulations were run with a time step size of Δ*t* = 5·10^−6^s on 256 Intel Xeon E5-2690 v3 CPU cores (*Piz Daint*, Cray XC40/50 supercomputer at the Swiss National Supercomputing Center CSCS) resulting in a wall time on the order of 5 days for the simulation of 0.3 s of physical time.

Instead of directly imposing pressure or velocity boundary conditions, we used periodic boundary conditions for the fluid domain. The flow through the AR was driven by a forcing term ***f***_TVPG_ which was added to the right-hand side of (1a),

(7) $$\begin{array}{l} f_{\text{TVPG}}\left(\text{x}\right)=\lambda \left(\text{x}\right)\cdot \left[\begin{array}{c} 100\cdot \left(0-v_{f,x}\right)\\ 100\cdot \left(0-v_{f,y}\right)\\ 8\cdot 133.3/0.006 \end{array}\right]\;\left[\text{Pa}/\text{m}\right] \end{array}$$

where λ(**x**) is equal to unity within a cylindrical domain of height 6 mm (magenta region in Figure 4 extending from *z* = −0.0277 m to *z* = −0.0217 m with radius *r*_*a*_ = 0.011 m) and zero otherwise. The *z*-component of this forcing term yielded a local pressure elevation of 8 mmHg which led to a flow through the valve with a mean TVPG of 8 mmHg. The *x*- and *y*-components of the forcing term (7) have the character of a fringe forcing which attenuated the inflow by penalizing lateral velocities. In summary, the forcing ***f***_TVPG_ fulfilled two roles: (a) creating a flow through the valve with a mean TVPG of 8 mmHg, (b) eliminating velocity fluctuations at the inflow (which may otherwise re-enter the AR from the outflow and through the periodic boundary conditions). However, we will see in the following, that the fringe forcing does not suppress all incoming velocity fluctuations such that residual fluctuations remain present in the inflow to the BHV.

### 2.4. Analysis of the Flow Field

The resulting flow field was analyzed for various instantaneous and statistical physical quantities. Table 1 gives an overview on the basic physical quantities used to characterize the flow through the BHV.

**Table 1:** Definition of physical quantities for flow characterization.

**Quantity**	**Symbol**	**Formula**
Aortic flow rate	*Q*(*t*)	∬*v*_*f, z*_(*x, y, z*_0_, *t*)d*x*d*y*
Jet area	*A*_jet_	∬*H*(*v*_*f, z*_(*x, y, z*_0_, *t*))d*x*d*y*
Equivalent jet radius	*r*_jet_	$\sqrt{A_{\text{jet}}/\pi }$
Average jet velocity	*v*_jet, avg_(*t*)	$A_{\text{jet}}^{-1}\iint v_{f,z}\left(x,y,z_{0}\right)H\left(v_{f,z}\left(x,y,z_{0},t\right)\right)\text{d}x\text{d}y$
Maximum jet velocity	*v*_jet, max_(*t*)	$\underset{A_{\text{jet}}}{\max}\{v_{f,z}\left(x,y,z_{0},t)\;\right\}$

*For the present configuration, these values are taken at z_0_=0.01 m which is slightly above the STJ. The integrals were taken over the AR lumen and H(·) is the Heaviside step function which is zero for negative arguments and unity for positive arguments*.

The computational model was configured to maintain a statistically steady flow through the valve model after an initial transient. This quasi-steady state was attained at *t* ≈ 0.2 s. This allowed us to compute the mean of a quantity *q*(*t*) as

(8) $$\begin{array}{l} \bar{q\left(t\right)}=\frac{1}{t_{2}-t_{1}}\int_{t_{1}}^{t_{2}}q\left(t^{\prime }\right)\text{d}t^{\prime }. \end{array}$$

For the present configuration, we used *t*_1_ = 0.2 s and *t*_2_ = 0.3 s.

For the analysis of the turbulent flow downstream of the BHV, the Reynolds decomposition of the flow field **v**_*f*_ yielded the mean flow field ${\text{V}}_{f}={\bar{\text{v}}}_{f}$ and the velocity fluctuations ${{\text{v}}^{\prime }}_{f}$ according to

(9) $$\begin{array}{l} {\text{v}}_{f}\left(\text{x},t\right)={\text{V}}_{f}\left(\text{x}\right)+{{\text{v}}^{\prime }}_{f}\left(\text{x},t\right) \end{array}$$

It will be shown in the following that the flow field comprised a periodic component related to vortex shedding at 40 Hz. To separate the coherent structures of this periodic component from the turbulent fluctuations, we performed a triple decomposition of the flow field according to Reynolds and Hussain (1972) which further decomposed the Reynolds fluctuation ${{\text{v}}^{\prime }}_{f}$ into a periodic component ${\tilde{\text{v}}}_{f}$ and a turbulent component ${{\text{v}}^{\prime \prime }}_{f}$,

(10) $$\begin{array}{l} {\text{v}}_{f}\left(\text{x},t\right)={\text{V}}_{f}\left(\text{x}\right)+{\tilde{\text{v}}}_{f}\left(\text{x},t\right)+{{\text{v}}^{\prime \prime }}_{f}\left(\text{x},t\right) \end{array}$$

The periodic component ${\tilde{\text{v}}}_{f}$ was computed as

(11) $$\begin{array}{l} {\tilde{\text{v}}}_{f}\left(\text{x},t\right)=\frac{1}{N}\overset{N}{\underset{j=1}{\sum }}{\text{v}}_{f}\left(\text{x},t+jT\right)-{\text{V}}_{f}\left(\text{x}\right) \end{array}$$

which includes a phase averaging the flow field over *N* periods of length *T*.

The magnitude of the fluctuations was characterized by the root-mean-square (mrs) of the fields ${{\text{v}}^{\prime }}_{f}$, ${\tilde{\text{v}}}_{f}$ and ${{\text{v}}^{\prime \prime }}_{f}$,

(12) $$\begin{array}{l} {v^{\prime }}_{\text{rms}}=\sqrt{\bar{{{\text{v}}^{\prime }}_{f}\cdot {{\text{v}}^{\prime }}_{f}}},\;{\tilde{v}}_{\text{rms}}=\sqrt{\bar{{{\tilde{\text{v}}}^{\prime }}_{f}\cdot {{\tilde{\text{v}}}^{\prime }}_{f}}},\;{v^{\prime \prime }}_{\text{rms}}=\sqrt{\bar{{{\text{v}}^{\prime \prime }}_{f}\cdot {{\text{v}}^{\prime \prime }}_{f}}}. \end{array}$$

By normalizing the rms values with the mean flow magnitude *V*_*f*_ = |**V**_*f*_|, we obtained turbulence intensities *I* and *I*″ and the intensity *Ĩ* of the periodic fluctuation,

(13) $$\begin{array}{l} I=\frac{{v^{\prime }}_{\text{rms}}}{V_{f}},\;\tilde{I}=\frac{{\tilde{v}}_{\text{rms}}}{V_{f}},\;I^{\prime \prime }=\frac{{v^{\prime \prime }}_{\text{rms}}}{V_{f}}. \end{array}$$

The shear strain rate and shear stress in the flow field were quantified via the strain rate tensor **S**,

(14) $$\begin{array}{l} \text{S}=\left\{S_{ij}\right\}=\frac{1}{2}\left[\nabla {\text{v}}_{f}+{\left(\nabla {\text{v}}_{f}\right)}^{T}\right]. \end{array}$$

The maximum instantaneous viscous shear stress was then computed as

(15) $$\begin{array}{l} \tau_{\text{max}}=\mu_{f}\left(s_{\text{max}}-s_{\text{min}}\right) \end{array}$$

where *s*_max_ and *s*_min_ are the maximum and minimum eigenvalues of **S**.

The Reynolds stress tensor was defined as

(16) $$\begin{array}{l} \Sigma_{ij}=-\rho_{f}\bar{{v^{\prime }}_{f,i}{v^{\prime }}_{f,j}} \end{array}$$

from which we derived a maximum Reynolds shear stress as

(17) $$\begin{array}{l} {\text{RSS}}_{\text{max}}=\sigma_{\text{max}}-\sigma_{\text{min}} \end{array}$$

where σ_max_ and σ_min_ are the maximum and minimum eigenvalues of {Σ_*ij*_}.

The turbulent dissipation rate ϵ was computed according to

(18) $$\begin{array}{l} \epsilon =2\nu_{f}\bar{S_{ij}S_{ij}} \end{array}$$

where ν_*f*_ = μ_*f*_/ρ_*f*_ is the kinematic viscosity.

One-dimensional velocity spectra *E*_*zz*_(*k*_*x*_) (Pope, 2000, p. 225) were computed from autocorrelation functions $R_{zz}\left(x^{\prime }\right)$ according to

(19) $$\begin{array}{l} E_{zz}\left(k_{x}\right)=\frac{1}{\pi }\int_{-\infty }^{\infty }R_{zz}\left(x^{\prime }\right){\text{e}}^{-\text{i}k_{x}x^{\prime }}\text{d}x^{\prime } \end{array}$$

(20) $$\begin{array}{r} R_{zz}\left(x^{\prime }\right)=\bar{{v^{\prime }}_{f,z}\left(x,y,z\right){v^{\prime }}_{f,z}\left(x+x^{\prime },y,z\right)} \end{array}$$

The wall shear stress **τ**_*w*_ acting on the aortic wall was computed as

(21) $$\begin{array}{l} \tau_{w}=2\mu_{f}\text{S}\text{n} \end{array}$$

where **n** was the unit normal vector of the wall pointing into the fluid domain. The magnitude of the wall shear stress was then given as

(22) $$\begin{array}{l} \tau_{w}=|\tau_{w}| \end{array}$$

from which we computed the time averaged wall shear stress as

(23) $$\begin{array}{l} \text{TAWSS}=\bar{\tau_{w}} \end{array}$$

The oscillatory and/or turbulent character of the wall shear stress is commonly quantified by the oscillatory shear index which was computed as

(24) $$\begin{array}{l} \text{OSI}=\left(1-|\bar{\tau_{w}}|/\bar{\tau_{w}}\right)/2 \end{array}$$

A value of OSI = 0 indicates unidirectional wall shear stress and 0.5 indicates oscillatory wall shear stress (Lee et al., 2009). Furthermore, we computed the time averaged magnitude of the WSS fluctuations as

(25) $$\begin{array}{l} \text{TAWSSF}=\bar{\left|\tau_{w}-\bar{\tau_{w}}\right|}. \end{array}$$

OSI and TAWSSF were used to characterize turbulent character of the wall shear stress.

## 3. Results

### 3.1. Transient Evolution of the Flow Field and Breakdown to Turbulence

**Figure 5:**
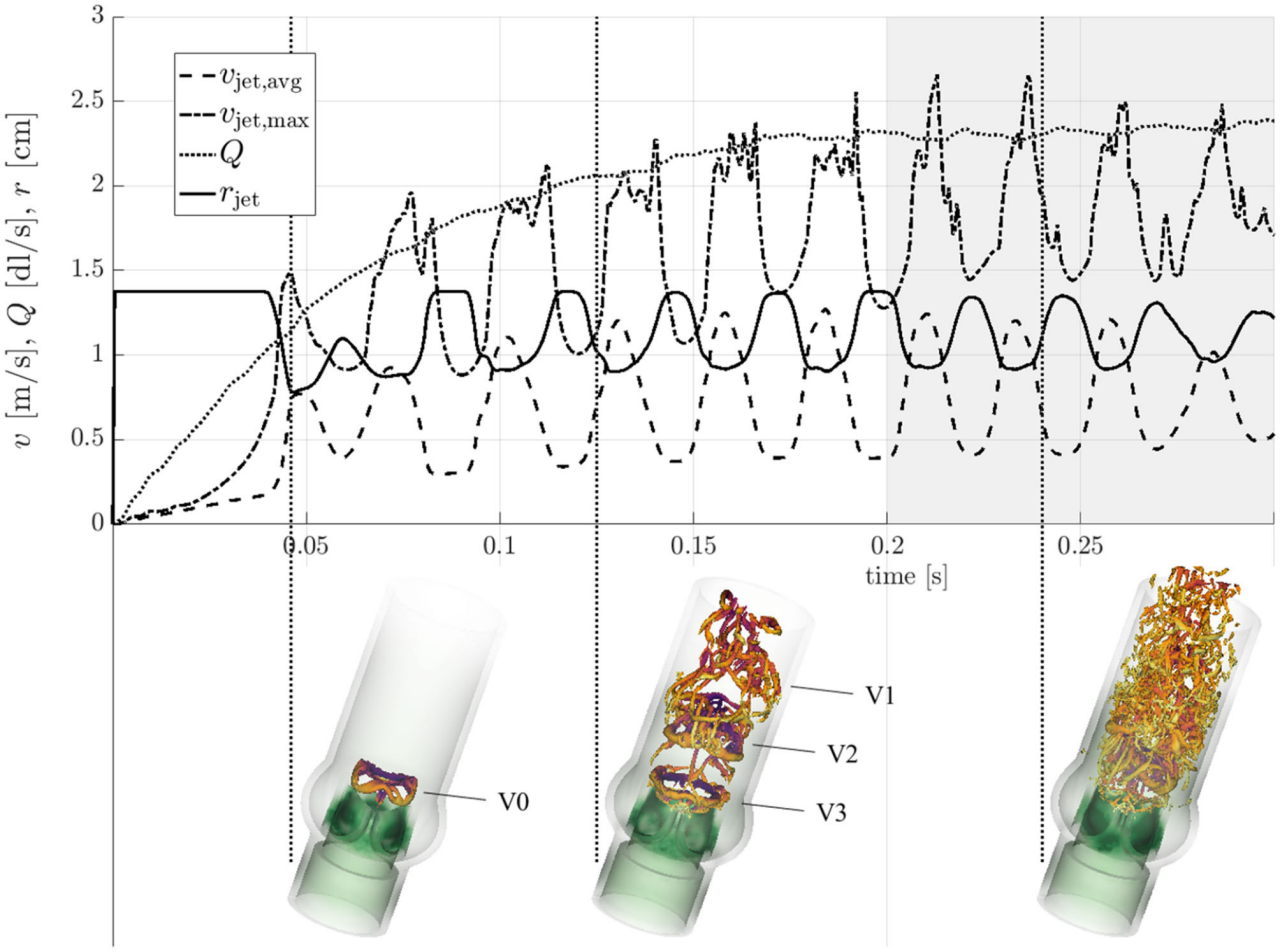
Transient evolution of the characteristic quantities form Table 1. Vortical structures (orange λ_2_-isosurfaces, Jeong and Hussain, 1995) are shown at time instances *t* = 0.046 s, 0.125 s, and 0.24 s (indicated by vertical dotted lines).

Figure 5 shows the evolution of the flow field from *t* = 0 to 0.3 s along with snapshots of vortical structures in the flow field at three distinct stages (see also the supplementary *flow field video* for an animated sequence of the flow field). At *t* = 0.046 s, immediately after valve opening, a starting vortex (V0 in Figure 5) was shed from the leaflet tips. This structured vortex ring was accompanied by a first peak of the average jet velocity *v*_jet, avg_. A similar three-lobed starting vortex was described in Sotiropoulos et al. (2016). Continued acceleration of the flow (increasing *Q* and *v*_jet, avg_, cf. Table 1) led to more complex vortex patterns. The second snapshot at *t* = 0.125 s highlights this development. At this point in time, the starting vortex V0 had already been advected out of the computational domain and three additional vortex rings (V1, V2, V3) had been shed from the valve orifice at regular intervals with a frequency close to 40 Hz (associated with peaks of *v*_jet, avg_). While the most recent vortex ring (V3 in Figure 5) was still an intact closed ring, the vortex ring V2 had been advected approximately one valve diameter downstream and was in the process of breaking down. Vortex V1 had already broken down completely to small scale vortical structures. After *t* = 0.2 s, the quantities in Figure 5, which were taken close to the valve orifice at *z* = 0.01 m, continued to show oscillations suggesting that there was continued shedding of vortex rings at 40 Hz. The flow field (third snapshot in Figure 5 taken at *t* = 0.24 s) exhibited a fully developed turbulent flow indicating rapid laminar-turbulent transition of the aortic jet and the vortex rings. This led to a Reynolds number for the flow through the AAo of Re_AAo_ = 2ρ_*f*_*Q*/(πμ_*f*_*r*_AAo_) ≈ 2000. The Reynolds number for the aortic jet was estimated as Re_jet_ = ρ_*f*_*v*_jet, avg_ *r*_jet_/μ_*f*_ ≈ 2100.

### 3.2. Leaflet Fluttering

**Figure 6:**
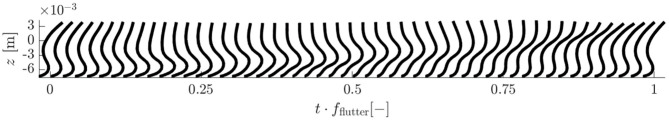
Cross-section of a leaflet through its symmetry plane from base to trailing edge. Its fluttering motion is shown throughout one period of its *f*_flutter_ = 40 Hz oscillation. The transversal deflections of the leaflet are shown to scale.

The vortex shedding at 40 Hz was directly connected to repeated transversal deflections of the valve leaflets which is also known as leaflet fluttering (Peacock, 1990). This fluttering showed amplitudes of several millimeters and had the same frequency *f*_flutter_ ≈ 40 Hz as the vortex shedding. Figure 6 depicts the leaflet motion throughout one fluttering period *T*_flutter_ = 1/*f*_flutter_ ≈ 0.025 s in a cross-section along its symmetry plane. The motion pattern had the form of a wave traveling from the base to the tip of the leaflet. The wave peak traveled at a mean velocity of approximately 0.4 m/s. The wave speed increased toward the end of the flutter period leading to a whiplash motion of the leaflet tip.

### 3.3. Mean Flow Field

**Figure 7:**
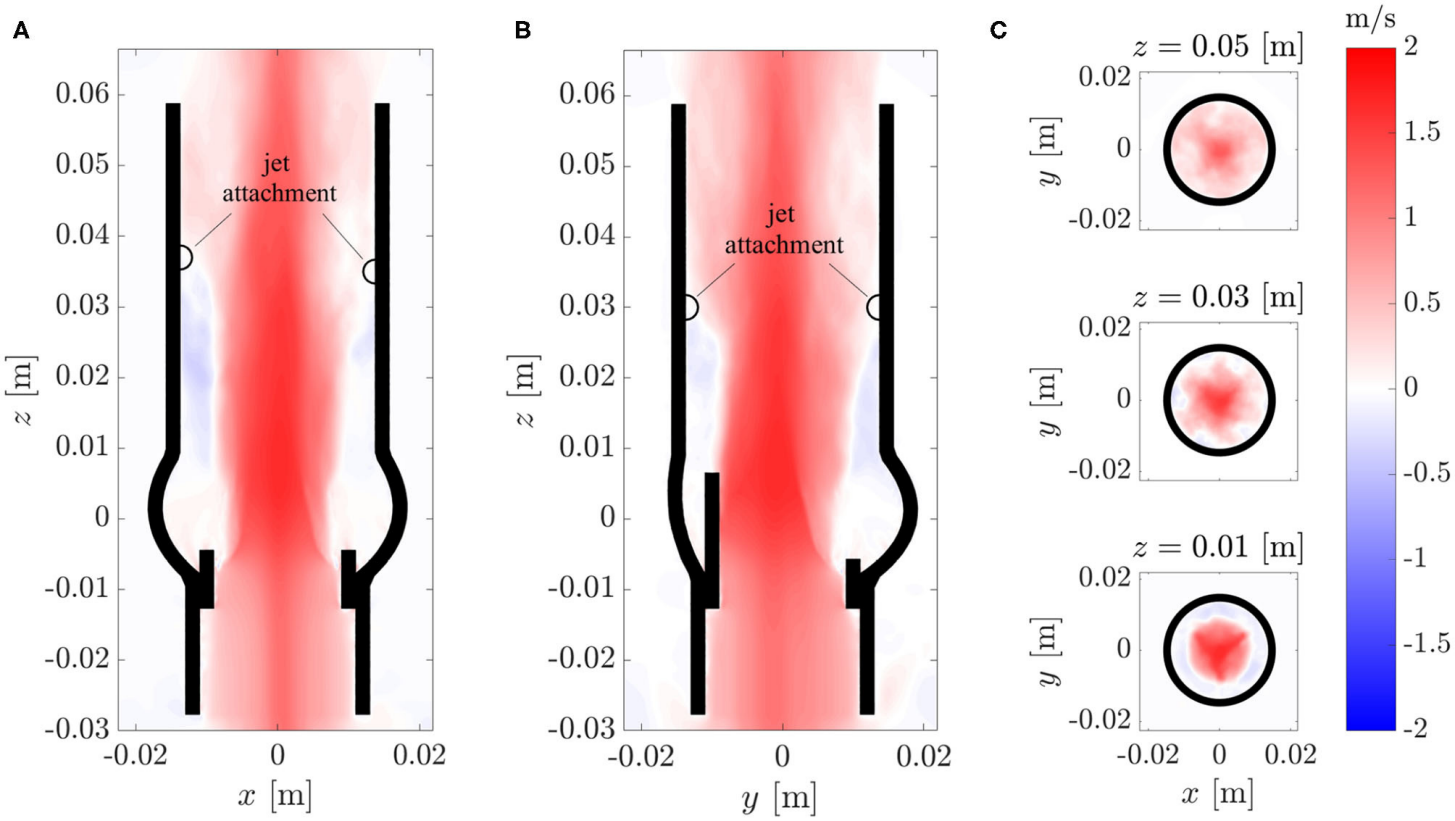
Axial component *V*_*z*_ of the mean flow field **V**_*f*_ in different cross-sections of the computational domains: **(A)** *xz*-plane, **(B)** *yz*-plane, **(C)** *xy*-plane.

The fully developed turbulent aortic jet in the center of the AR showed mean velocities beyond 2 m/s. The axial component *V*_*f, z*_ of the mean flow is depicted in Figure 7 for various cross-sections cutting the domain centrally in the *xz*-plane and *yz*-plane and at different *xy*-planes. Besides the central aortic jet, there were retrograde flow regions between the AAo wall and the aortic jet. These regions started about one valve diameter downstream of the BHV (*z* ≈ 0.03…0.04 m), where the aortic jet attached to the AAo wall, and transported fluid back toward the sinus portions.

The cross-sections in Figure 7C show that the aortic jet was not axisymmetric. At *z* = 0.01 m, it featured a star-shaped region with higher velocities. The three points of the star were aligned with the valve posts and commissures. At *z* = 0.03 m, the cross-section resembled a six-pointed star which is the reason why the attachment points of the jet in th *xz*-plane (Figure 7A) were farther downstream than the attachment points in the *yz*-plane (Figure 7B). At *z* = 0.05 m, these features had mostly disappeared and the character of the mean flow field had transitioned from a free jet to a pipe flow.

### 3.4. Turbulent Flow Field

**Figure 8:**
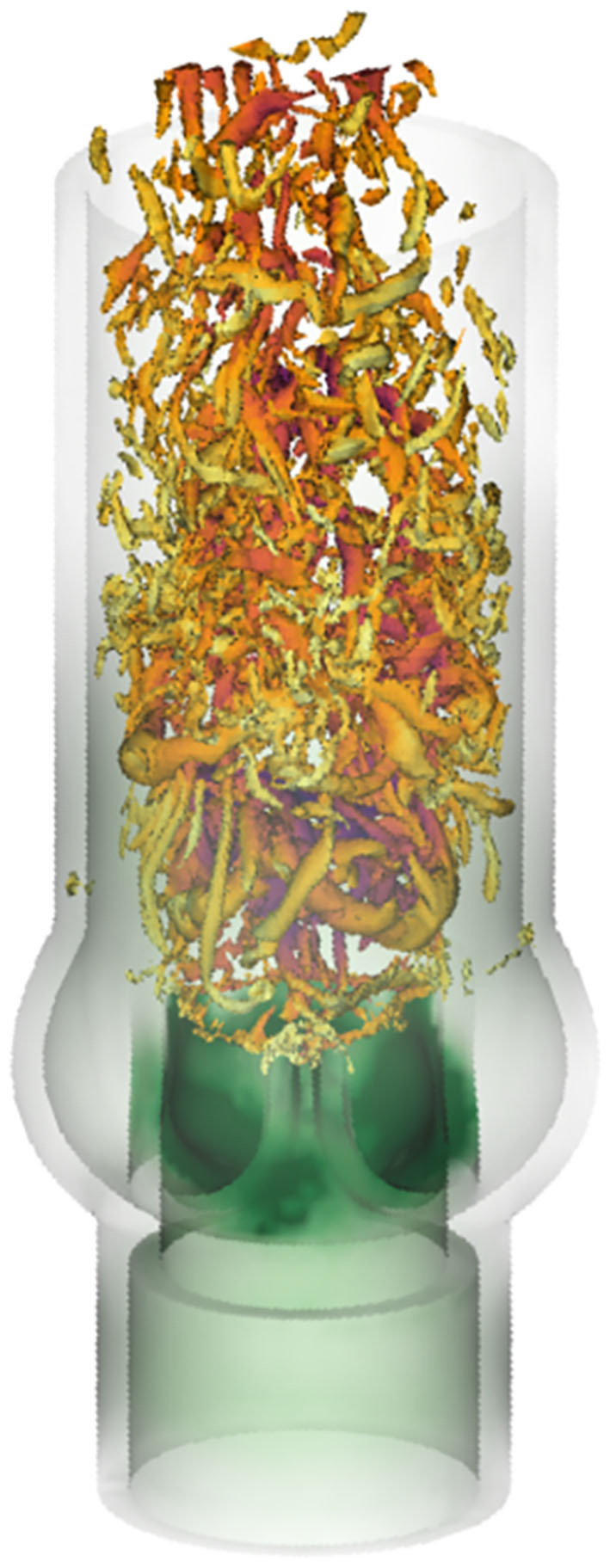
Vortical structures at *t* = 0.24 s visualized by isosurfaces of the λ_2_-criterion by Jeong and Hussain (1995).

The breakdown of the aortic jet into small eddies is illustrated in Figure 8 and in the supplementary *flow field video*. The vortex rings, which were issued at 40 Hz, broke down rapidly into turbulent structures. Visual inspection of Figure 8 suggests that the flow field was most chaotic approximately one valve diameter downstream of the orifice. Farther downstream, the turbulent flow field appeared to calm down. These qualitative observations are in line with experimental observations shown in Figure 1 and we will demonstrate in the following that they can be substantiated quantitatively.

**Figure 9:**
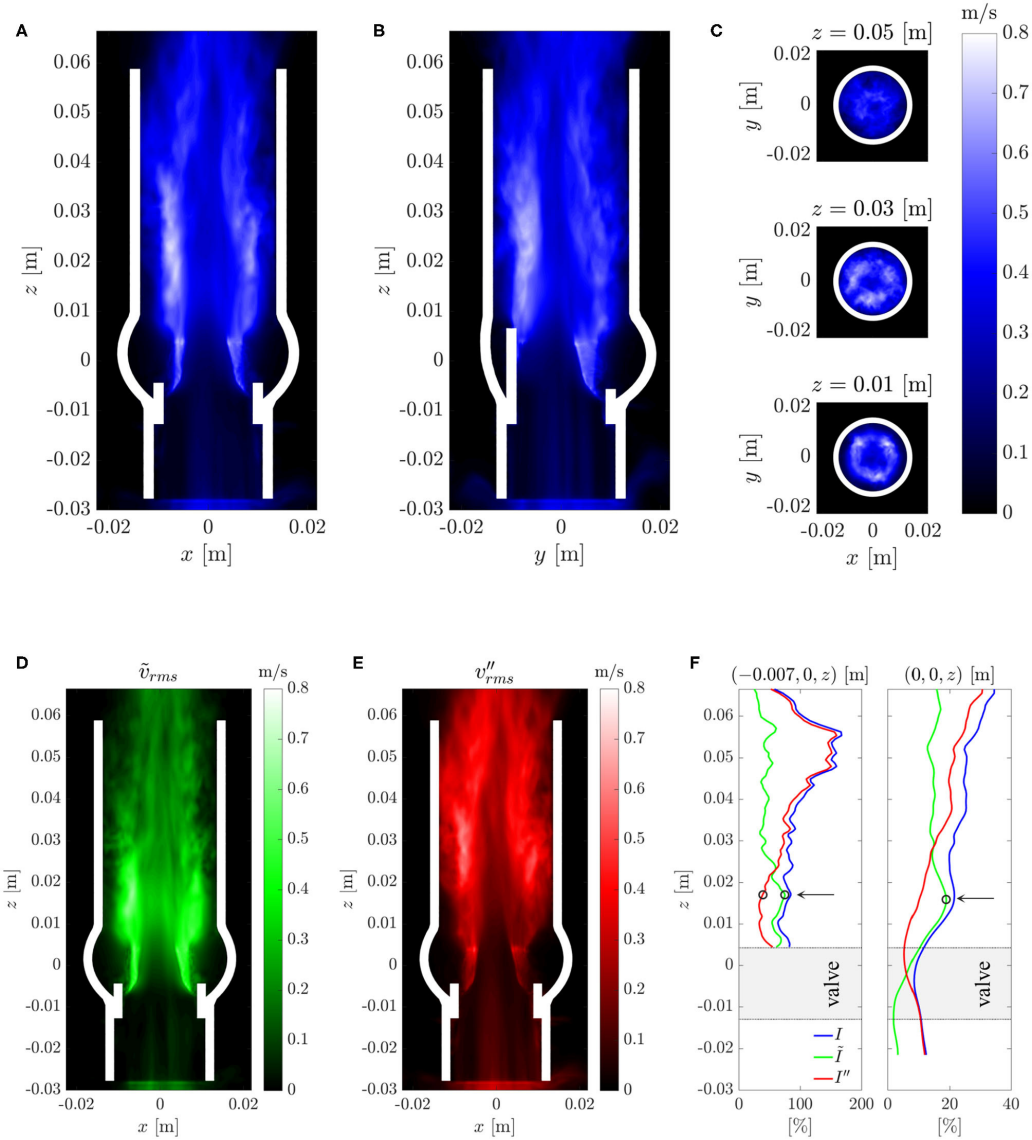
**(A–C)** Root-mean-square velocity fluctuations ${v^{\prime }}_{\text{rms}}$ in different cross-sections (same as in Figure 7). **(D)** Root-mean-square periodic fluctuations *ṽ*_rms_ and **(E)** root-mean-square turbulent fluctuations ${v^{\prime \prime }}_{\text{rms}}$. **(F)** Turbulent intensities *I* and *I*″ and the intensity *Ĩ* of the periodic fluctuations along an axial line in the shear layer (left) and along the centerline (right). The arrows and circles indicate the position of the peak intensity *Ĩ* of the periodic fluctuations and the begin of the growth of the turbulence intensity *I*″ within the shear layer.

Figures 9A–C shows the root-mean-square (rms) fluctuation field ${v^{\prime }}_{\text{rms}}$ in the same cross-sectional planes as in Figure 7. The highest rms values were found above the valve posts which coincided with the orientation of the tree-pointed star within the central jet. Therefore, the highest fluctuation amplitudes were found in regions where the mean flow featured the sharpest shear layers. The center of the aortic jet showed very little velocity fluctuations as it is common for potential cores of jets. With increasing downstream distance, the fluctuation field became more homogeneous and had a reduced amplitude suggesting decaying turbulent intensity and increasingly homogeneous turbulent flow.

The triple decomposition of the flow field, Equation (10), was computed to better discriminate between the coherent vortex structures shed from the BHV at a frequency of 40 Hz and the turbulent fluctuations. To this end, the flow field between *t* = 0.2 s and 0.3 s was phase averaged over four fluttering periods of *T*_flutter_ = 0.025 s according to Equation (11). The rms values for the periodic fluctations ${\tilde{\text{v}}}_{f}$ and for the turbulent fluctuations ${{\text{v}}^{\prime \prime }}_{f}$ (Figures 9D,E) indicate that the periodic fluctuations dominated the flow between the valve and *z* ≈ 0.025 m whereas the turbulent fluctuations dominated the flow field for *z* > 0.025 m. Figure 9F shows the turbulence intensities *I* and *I*″ and the intensity *Ĩ* of the periodic fluctuations along the centerline and along an axial line (−0.007 m, 0 m, *z*) which passed through the shear layer. This data confirms the observations from above and shows that the intensity *Ĩ* of the periodic fluctuations had a distinct peak at *z* ≈ 0.015 m. This peak also marks the point where the turbulence intensity *I*″ in the shear layer began to increase. Further, we find that the turbulence intensity at the inflow (after the fringe region) was approximately 10% which indicates that the fringe region did not suppress all fluctuations. However, we also observe that the turbulence intensity *I*″ on the centerline decreased to 5% within the valve suggesting that the residual turbulent fluctuations were attenuated in the accelerated flow through the valve. The turbulence intensity *I*″ increased again beyond 10% only after the peak of *Ĩ* at *z* ≈ 0.015 m.

### 3.5. Viscous Shear Stress and Reynolds Shear Stress

**Figure 10:**
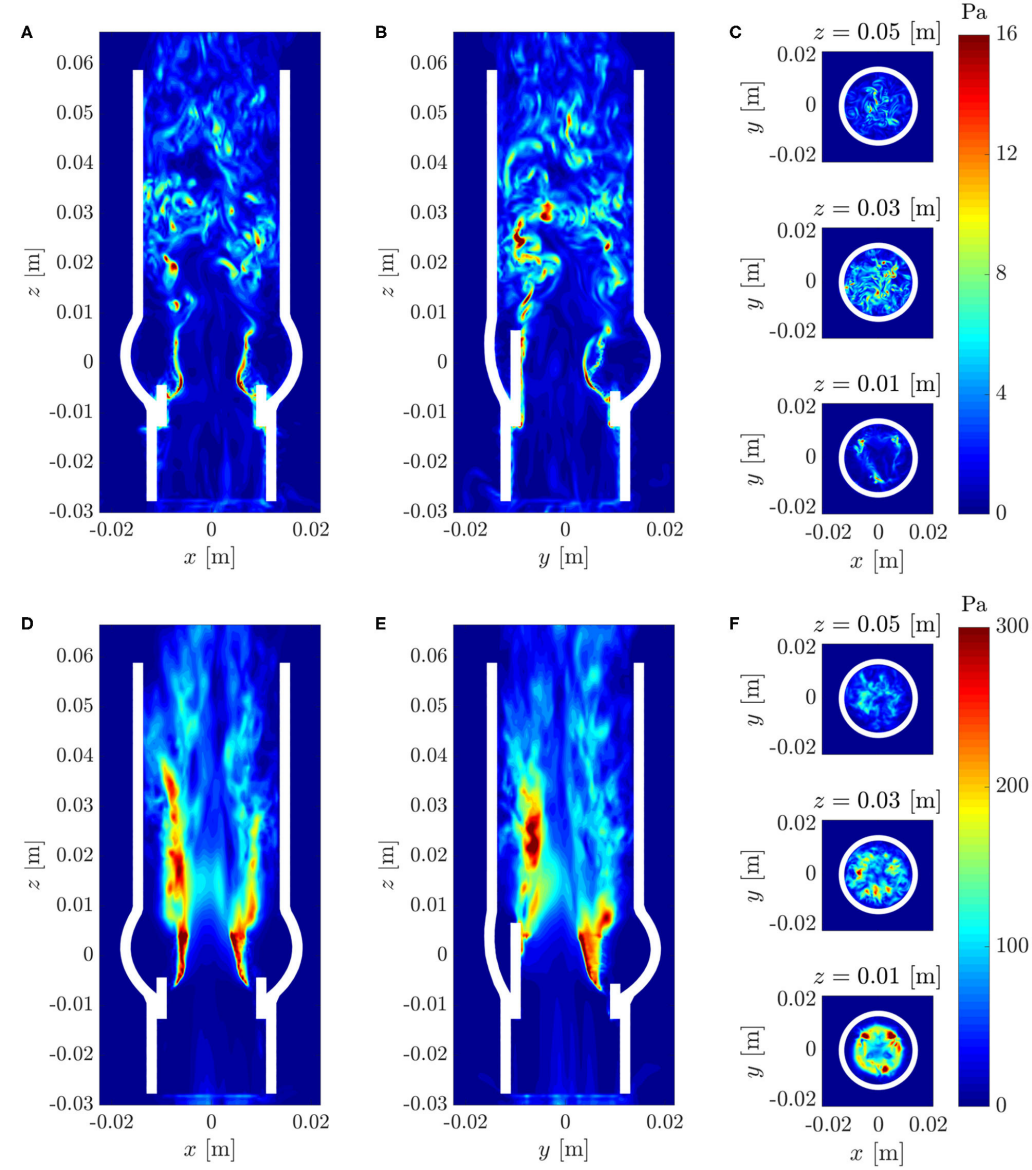
**(A–C)** Instantaneous maximum viscous shear stress τ_max_ at *t* = 0.3 s; **(D–F)** Maximum Reynolds shear stress RSS_max_ (same cross-sections as in Figure 7).

Maximum viscous shear stresses τ_max_ (15) were computed for the flow field at *t* = 0.3 s (Figures 10A–C). The resulting stress field was consistent with the above observations in that it showed peak values at a height of *z* ≈ 0.03 m. The colorbar is set to an upper limit of 16 Pa but peak values within the whole domain reached up to 40 Pa.

Figures 10D–F shows the maximum Reynolds shear stress RSS_max_ (17). The regions of high RSS_max_ at the location of the leaflets were artifacts due to the immersed boundary implementation of the fluid-structure interaction and should not be considered for the analysis of the turbulent flow field. Otherwise, the Reynolds stress field showed a similar structure as the rms velocity fluctuations (Figure 9) and exhibited values up to 300 Pa within the shear layers and above the valve posts.

### 3.6. Turbulent Dissipation Rate

**Figure 11:**
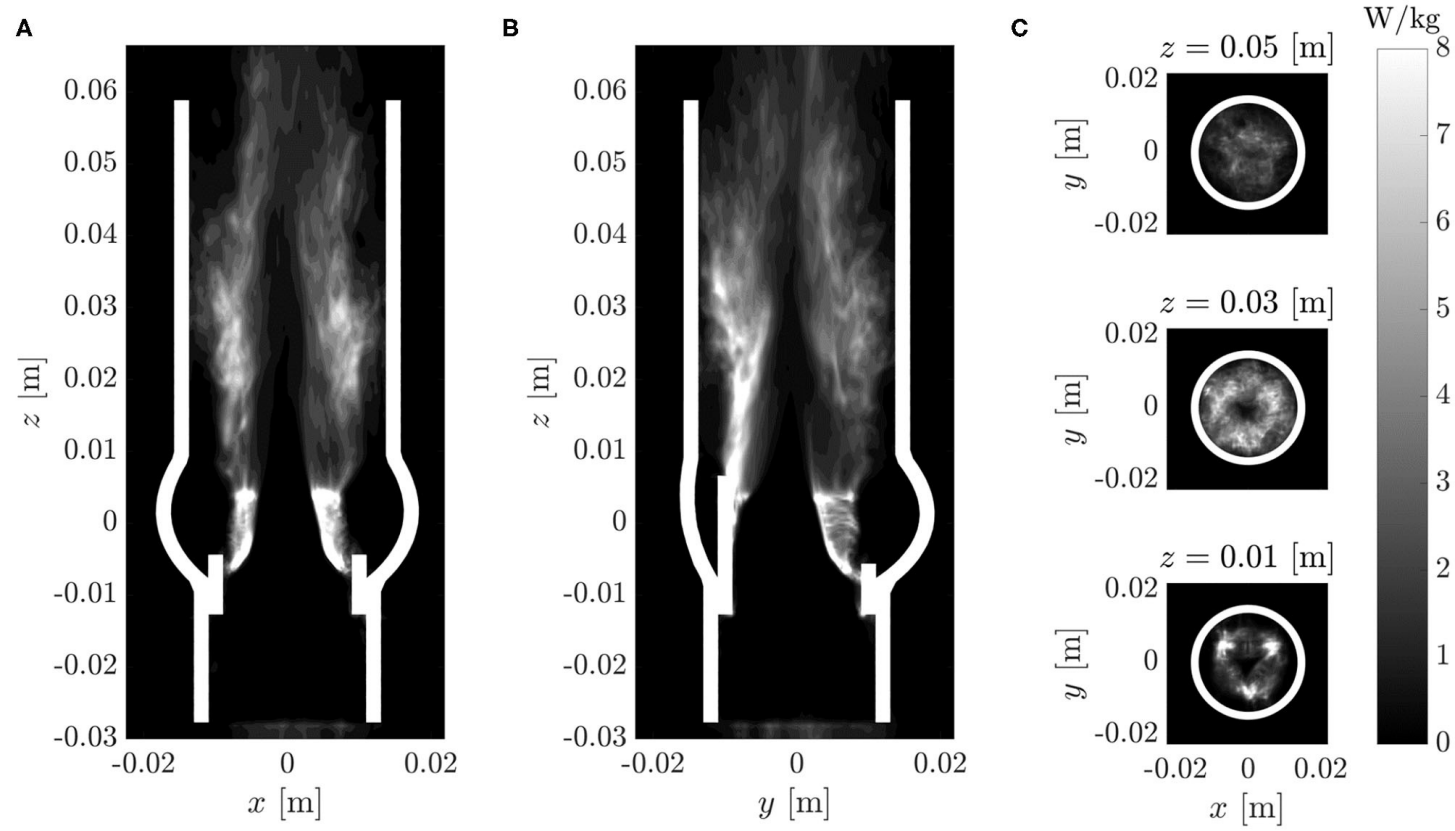
Turbulent dissipation rate ϵ (same cross-sections as in Figure 7).

The turbulent dissipation rate ϵ (18) is illustrated in Figure 11. Consistent with the distribution of the rms fluctuations (Figure 9), the highest dissipation rates were in the shear layers above the valve posts. Beyond *z* = 0.03 m, the dissipation rates decreased in amplitude. There was an almost dissipation-free region in the jet core which closed at *z* ≈ 0.05 m.

Similar to the plots for RSS_max_, Figures 11A,B include artifacts due to the immersed leaflet structures. Although the dissipation rates at these locations are physically not meaningful, they visualize the extent of the leaflet excursions during multiple fluttering periods.

**Figure 12:**
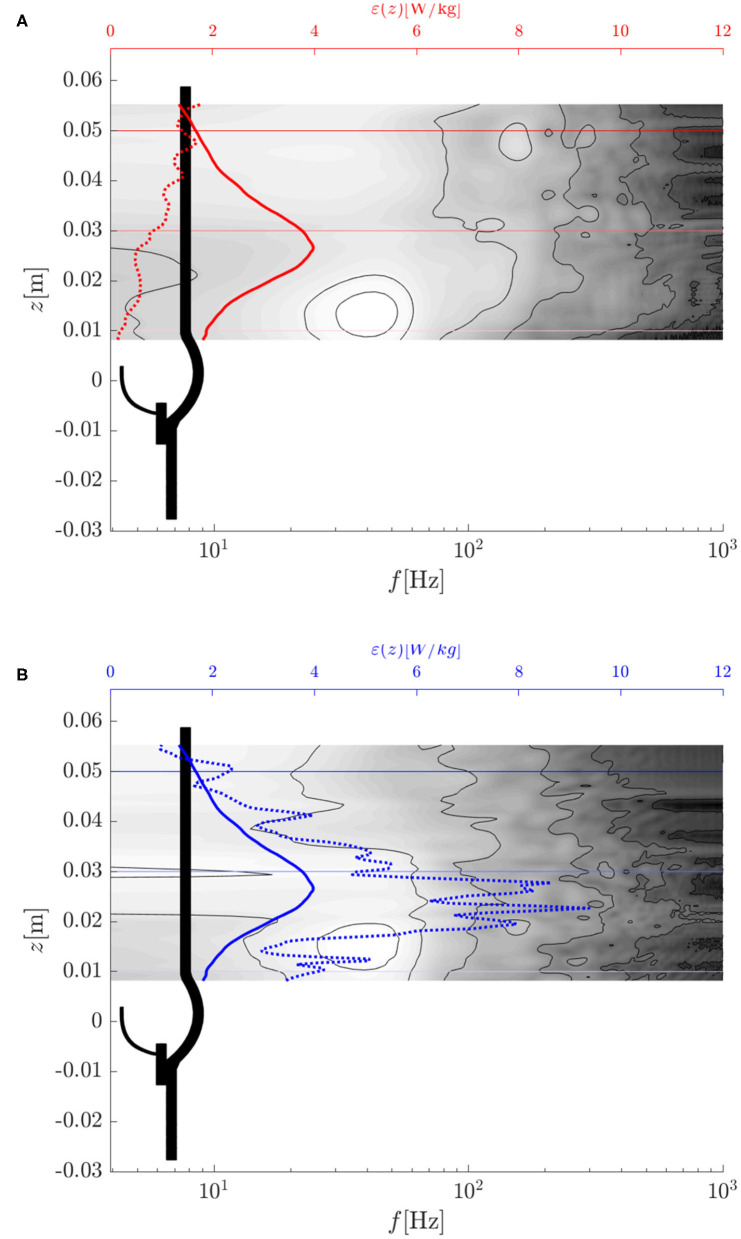
Turbulent dissipation rate ϵ (dotted lines), average turbulent dissipation rate ϵ_avg_ (solid lines) and temporal power spectral density of ${v^{\prime }}_{f,z}\left(x_{0},y_{0},z\right)$ (contour plot: white indicates high values, dark for low values): **(A)** along the center line (*x*_0_, *y*_0_, *z*) = (0, 0, *z*) m, **(B)** along a parallel line (*x*_0_, *y*_0_, *z*) = (−0.007, 0, *z*) m.

Figure 12 shows the evolution of the energy dissipation rate ϵ along the centerline of the AR (dotted red curve) and along a parallel line (dotted blue curve) which passed through the shear layer at the edge of the aortic jet at {*x*_0_, *y*_0_, *z*} = {−0.007 m, 0 m, *z*}. Whereas, the dissipation rate along the centerline was very small at the valve orifice and increased in the downstream direction, the dissipation rate along the shear layer had a peak between *z* = 0.02 and 0.03 m after which it decayed toward the value of the centerline dissipation rate.

The average dissipation rate ϵ_avg_ (average over the cross-section at the given height) is shown in Figure 12 by solid blue and red lines (the two curves are identical). Evidently, the average dissipation rate reached a distinct peak at *z* ≈ 0.027 m after which it decreased monotonically. This suggests that the flow experienced the most intense turbulent dissipation approximately one valve diameter past the valve orifice which is in line with our previous observations.

The total turbulent dissipation rate in the AAo was

(26) $$\begin{array}{l} P_{\text{turb}}=\rho \int_{V}\epsilon \text{d}V=0.064\text{W} \end{array}$$

where the integral was evaluated over the AAo volume from *z* = 0.01 m to *z* = 0.05 m. This turbulent dissipation rate can be compared to the rate of mechanical energy loss of the flow through the BHV which amounted to *P*_mech_ = Δ*p*_mean_·*Q*_mean_ = 0.245 W, where Δ*p*_mean_ is the mean TVPG. Comparison of the two values shows that 26% of the total mechanical losses can be attributed to turbulence. Or in other words, approximately 2.5 mmHg of the TVPG of 8 mmHg were due to turbulent flow.

### 3.7. Velocity Spectra

Spectra of the velocity fluctuations were studied at different locations. To this end, the power spectral density of time series of velocity fluctuations were computed with MATLAB's pwelch function. Figure 12 shows the power spectral density of the axial velocity fluctuation ${v^{\prime }}_{f,z}$ sampled at different heights *z* on the centerline and on a parallel line passing through the shear layer of the jet.

In both data sets, the leaflet fluttering frequency *f*_flutter_ ≈ 40 Hz dominated the spectra close to the valve orifice (*z* = 0.01…0.02 m). These peaks can be associated with the vortex shedding from the valve orifice. Farther downstream, the peaks vanished while broad-banded spectra were established. This process was completed at *z* ≈ 0.03 m where the average turbulent dissipation rate reached its peak value.

**Figure 13:**
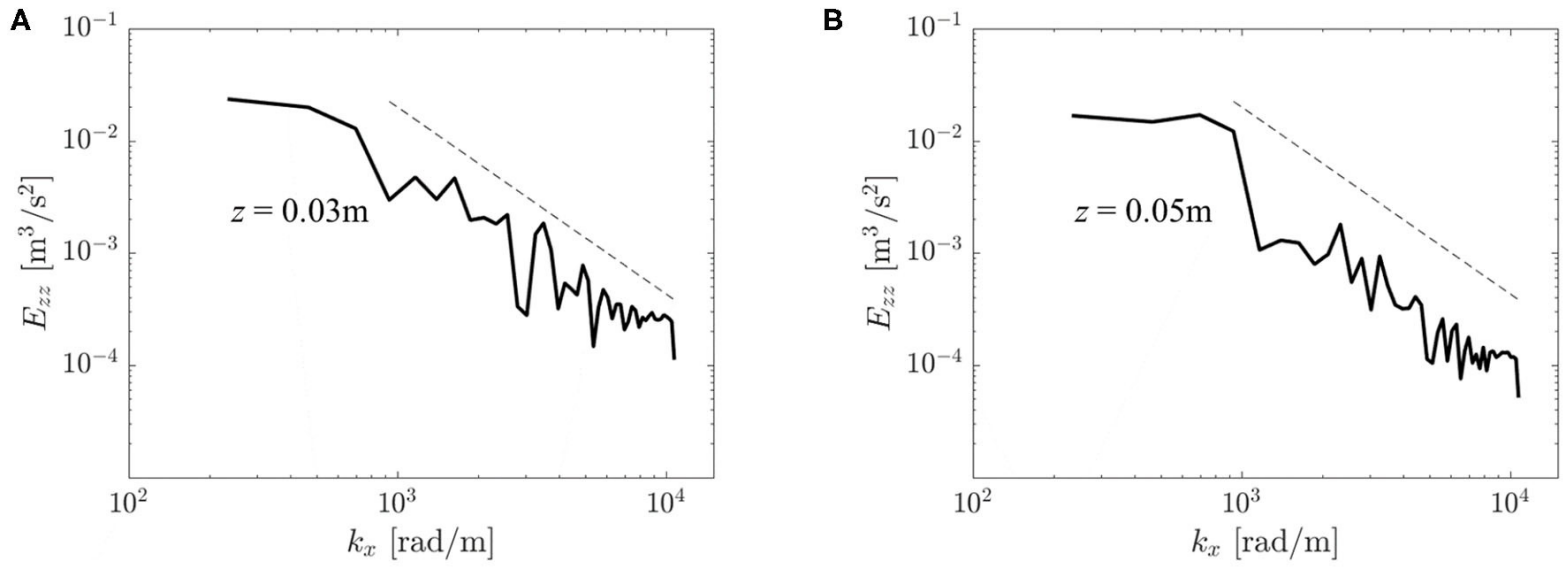
Velocity spectra *E*_*zz*_(*k*_*x*_) at different locations in the flow field: **(A)** (*x, y, z*) = (0, 0, 0.03) m, **(B)** (*x, y, z*) = (0, 0, 0.05) m. The dashed line shows a −5/3 decay rate typical for a turbulent inertial subrange.

Figure 13 shows two velocity spectra *E*_*zz*_ of the turbulent flow field. They were obtained from autocorrelation functions $R_{zz}\left(x^{\prime }\right)$ for two points on the centerline at *z* = 0.03 and *z* = 0.05 m. Both spectra comprised a wavenumber range with −5/3-slope which is reminiscent of a turbulent inertial subrange (Pope, 2000, p. 230). At *z* = 0.05 m, the energy in this range was somewhat lower than at *z* = 0.03 m indicating a decay in turbulent intensity in downstream direction.

### 3.8. Wall Shear Stresses

**Figure 14:**
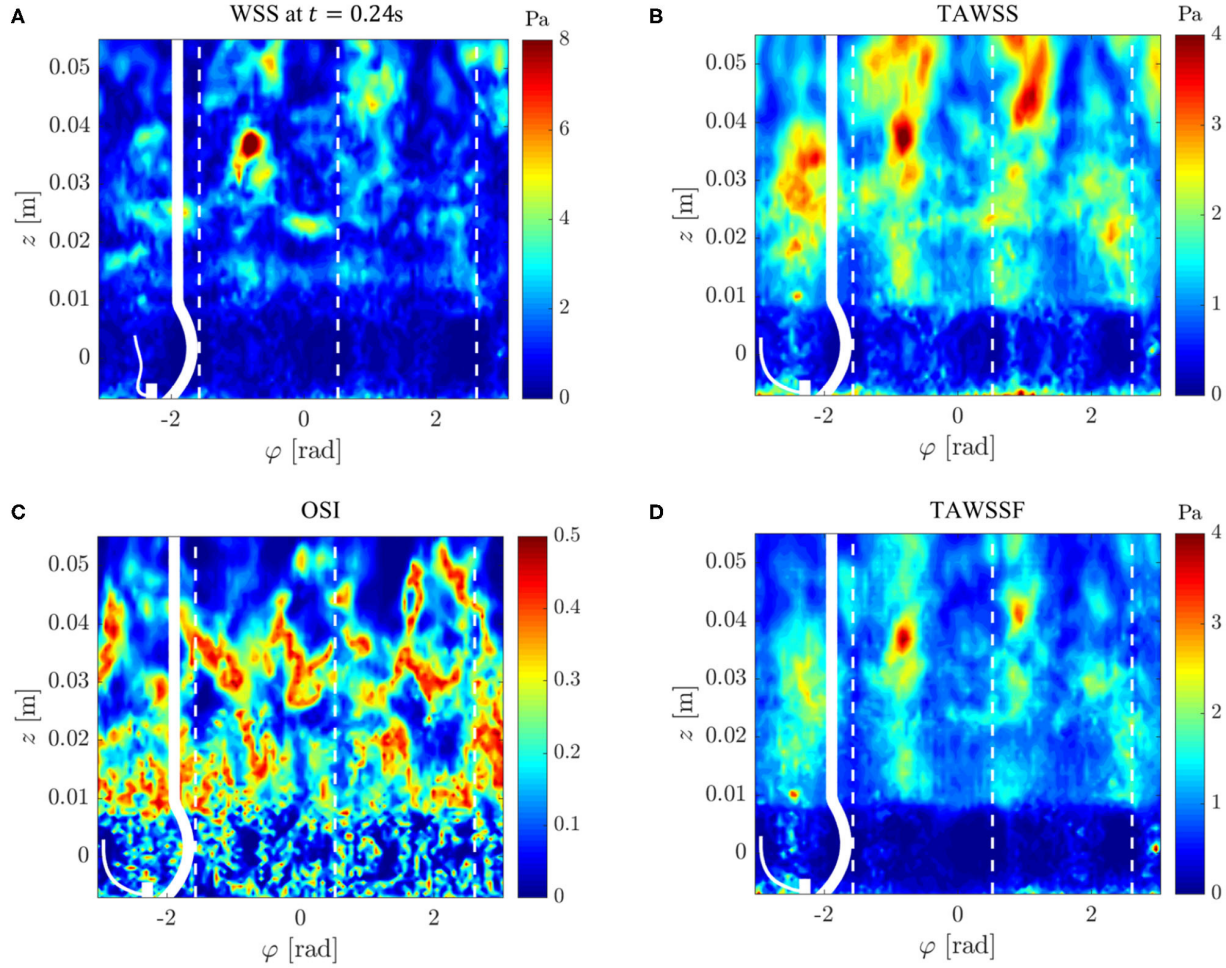
Wall shear stresses on the AR wall: **(A)** instantaneous WSS at *t* = 0.24 s, **(B)** mean WSS, **(C)** oscillating shear index OSI, **(D)** mean magnitude of WSS fluctuations. The values were projected onto an unrolled cylinder surface where the vertical dashed lines highlight the azimuthal locations of the valve posts.

Figure 14A illustrates the WSS distribution for the fully developed flow at *t* = 0.24 s. We found irregular WSS patterns mainly downstream of the STJ with peak values beyond 12 Pa. *WSS video* (Supplementary Material) shows the temporal evolution of WSS throughout the whole simulation. This video also shows repeated bands of elevated WSS between *z* = 0.01 and 0.02 m which were traveling in downstream direction. These bands were most likely footprints of the vortex rings shed from the valve orifice.

The mean WSS (Figure 14B) exhibited values mostly below 3 Pa. This value is roughly four times higher than the WSS of a Poiseuille flow at equivalent flow rate in a pipe with radius *r*_AAo_. Figures 14C,D illustrate the fluctuating character of the WSS. The high oscillating shear index OSI highlights the unsteady character of the flow above the STJ, whereas the OSI in the sinus portions was closer to zero indicating rather steady flow patterns. The mean magnitudes of the WSS fluctuations (Figure 14D) support this observation: there were no WSS fluctuations of notable magnitude in the sinus portions (below *z* = 0.01 m). At the same time, the WSS fluctuations were significantly higher downstream of the STJ and reached maximum values of approximately 4 Pa at *z* ≈ 0.04 m.

We conclude from these observations that the turbulent systolic flow led to high WSS levels. Highest WSS fluctuations were noted beyond *z* = 0.03 m where the free aortic jet attached to the AAo wall (cf. Figure 7). Finally, it should be pointed out that (somewhat weaker) turbulent WSS patterns were also identified between *z* = 0.01 and 0.03 m where the flow field close to the wall was dominated by retrograde flow.

## 4. Discussion

It was the primary aim of this study to characterize the turbulent systolic flow of a BHV. To this end, we designed a computational model of a BHV in an anatomically correct model of an AR. The governing equations were solved with a FSI solver (Nestola et al., 2019) which comprises a finite-element structural solver for soft tissue and a high-order Navier–Stokes solver that has been designed for the study of laminar-turbulent transition (e.g., Obrist et al., 2012; John et al., 2016). The FSI solver was verified and validated for canonical benchmarks in Nestola et al. (2019). For validation of the present model, we will show in the following that the computational model yielded results which were consistent with experimental observations. In particular, we will compare our results to experimental results by Hasler and Obrist (2018) and Vennemann et al. (2018) who studied the same bovine BHV as in the present work.

The topology of the mean flow with a central aortic jet with maximum velocities of approximately 2.5 m/s and retrograde flow at approximately 0.5 m/s along the AAo wall (Figure 7) agrees very well with the experimental observations of Hasler and Obrist (2018). These observations are also consistent with the instantaneous flow field at peak systole reported by Lee et al. (2020) which was obtained with a computational model for a similar BHV configuration and which exhibited the same flow topology with a non-axisymmetric central jet and retrograde flow along the AAo wall.

For further validation of the computational results against experimental data, selected results from the study of Hasler and Obrist (2018) are shown in the Supplementary Material (*PIV data*) where the experimental data were processed and plotted in the same way as the results of the present study. Inspection of this data shows that there is also good agreement between experimental and computational data for the turbulent flow fields: The rms fluctuation velocities (Figure 9 and Supplementary Figure 1) show the same maximum amplitudes of approximately 0.8 m/s in the shear layers of the aortic jet at *z* ≈ 0.03 m. Likewise, RSS_max_ (Figures 10D–F and Supplementary Figure 2) peak in the same region at approximately 300 Pa. The concentration of rms fluctuations and RSS above the valve posts could not be observed in the experiment. We suspect that this difference was due to different inflow conditions in experiment and simulation. It is possible that higher inflow disturbance levels in the experiment led to a less organized flow field which made it more difficult to identify local structures above the valve posts. Finally, Supplementary Figure 3 shows velocity spectra *E*_*zz*_(*k*_*x*_) for the same locations as the spectra in Figure 13. Although these spectra exhibit the same overall structure as the computational data, the experimental velocity spectrum at *z* = 0.03 m (Supplementary Figure 3A) shows a significantly lower energy level than the computational data. This may be attributed to the laminar core which had not yet fully closed at *z* = 0.03 m in the experiment. At *z* = 0.05 m, the laminar core had closed in the experiment and in the computational model and the turbulent flow field attained a more homogeneous character. Accordingly, the experiential velocity spectrum at *z* = 0.05 m (Supplementary Figure 3B) is in very good agreement with the computational data.

The leaflet fluttering at 40 Hz agrees very well with observations of Vennemann et al. (2018) who described leaflet fluttering at 36 Hz in an experiment with similar hemodynamic configuration and the same valve design. The present results are also close to the experimental observations of Peacock (1990) who reported fluttering at 15 to 30 Hz for a different bovine BHV in water. Recent *in vivo* electrocardiographic measurements in stentless bovine BHV found fluttering frequencies of 15 Hz (Aljalloud et al., 2018). Lee et al. (2020) reported fluttering of a bovine BHV in a pulse duplicator in saline at approximately 30 Hz while their numerical model exhibited fluttering at approximately 60 Hz (estimated from Figure 4 in Lee et al., 2020). These comparisons suggest that the present model was appropriate to reproduce the phenomenon of leaflet fluttering, although the fluttering frequency was somewhat higher than in corresponding experimental settings. Quantitative differences to the experimental results of Moore and Dasi (2014), who observed fluttering at 50−100 Hz for a porcine BHV in saline and no fluttering when the a water/glycerin mixture was used, may be due to the porcine tissue with results in thinner and more supple leaflet structures than in bovine BHV. The amplitude of the fluttering appears rather high when compared to experimental data. Whereas, the leaflet tips in the present study moved approximately 5 mm during a fluttering period, Peacock (1990) reports amplitudes of only 2 mm and Moore and Dasi (2014) observed fluttering amplitudes of 4 mm. It is not clear at this point, what causes these large amplitudes and further studies are necessary to understand which mechanical or geometrical parameters determine the fluttering amplitude.

We showed that leaflet fluttering was directly connected to repeated shedding of vortex rings at 40 Hz. These vortex rings are an inherent feature of the fully developed aortic jet and must not be confused with the starting vortex which is only issued once after valve opening (Sotiropoulos et al., 2016). The triple decomposition of the flow field (Figures 9D–F) suggests that the fluctuations due to the vortex rings had a peak at *z* ≈ 0.015 m after which the turbulence intensity *I*″ started to increase, marking the start of the vortex ring breakdown. This process was also reflected in the temporal spectra of Figure 12 which were dominated close to the valve orifice (at *z* = 0.01 m) by the shedding frequency of 40 Hz and then evolved to turbulent spectra featuring the classical −5/3-slope of the inertial subrange (Figure 13). The peak in turbulent dissipation at *z* ≈ 0.03 m suggested that this region may have been the place of turbulent breakdown. This region was also associated with the highest viscous shear stresses and Reynolds shear stresses (Figure 10).

While RSS levels are of limited relevance for the prediction of blood trauma (Quinlan and Dooley, 2007; Ge et al., 2008), the viscous shear stress levels (up to 40 Pa) indicate that shear-induced thrombocyte activation may occur in the shear layers of the aortic jet. As already pointed out by Hedayat et al. (2017) and Hedayat and Borazjani (2019) for the case of bileaflet MHV, this activation in the aortic jet may become clinically relevant, if activated thrombocytes are transported by the retrograde flow along the wall toward the sinus portions where thrombi may form (Chakravarty et al., 2017; Jahren et al., 2018; Hatoum et al., 2019).

The turbulent flow behind the BHV left its footprint also on the AAo wall. We found elevated and fluctuating WSS up to 14 Pa mainly downstream of the point where the aortic jet attached to the wall (Figure 14). These turbulent WSS may play a role in lesions of the endothelial layer (Davies et al., 1986). The present results also indicate that the distribution and intensity of turbulent WSS on the AAo wall are related to the point of attachment of the central aortic jet. This suggests that the ratio between aortic jet diameter and AAo diameter could be relevant for the location and magnitude of peak WSS: the larger the AAo diameter, the later the attachment and the lower and farther downstream the peak WSS. Of course, these conjectures must be confirmed quantitatively and it should also be pointed out that the present model with a straight AAo did not reflect the full geometrical complexity with the bending of the AAo which may lead to an early impingement (rather than an attachment) of the aortic jet.

Further limitations of the present study include the inflow, the modeling of the BHV and the high fluid viscosity used in our model. As we have seen in Figure 9F, the inflow to the BHV had a residual turbulence intensity *I* of approximately 10% due to incomplete suppression of fluctuations in the fringe region. It is unclear whether this level of fluctuations is a good representation of the inflow to a BHV coming from the left ventricle or the inflow in an experimental setup. It can be expected that the complex and unsteady flow within the left ventricle will lead to significant inflow disturbances. However, their magnitude is not known and further studies are necessary to assess to what extent the inflow perturbations affect the transition process behind the valve.

The BHV was modeled using manual measurements of the valve dimensions and literature data on the mechanical properties of bovine pericard tissue used for the valve leaflets. Although the valve kinematics matches experimental observations generally quite well, the high fluttering amplitude suggests that the present BHV model should be further refined by optimizing mechanical and geometrical parameters of the model.

To assess the effect of the fluid viscosity on the results, we performed *ad hoc* simulations with a simplified model with viscosities of 0.004, 0.006, and 0.008 Pa s. These tests indicated that the viscosity had a negligible effect on the mean flow field and only a very small effect on the location of the turbulent breakdown. For the lower viscosity (0.004 Pa s), we found an increase of 10% of the turbulent kinetic energy with respect to the nominal viscosity (0.006 Pa s) and an decrease of 5% for the highest viscosity (0.008 Pa s). The average viscous stresses changed nearly proportionally to the viscosity which also indicates that the shear rates remained nearly unchanged. Therefore, it should be expected that the viscous shear stress levels and the wall shear stress levels for blood are reduced by 30 to 40%.

Finally, we would like to discuss differences between the pulsatile flow through a real heart valve and the quasi-steady systolic flow configuration in the present study. Certainly, the present configuration eliminated some transient effects due to flow pulsatility and we found that fluctuations of *r*_jet_, *v*_jet, avg_, and *v*_jet, max_ slightly decayed toward the end of our simulation (cf. Figure 5). This could indicate that the fluttering is not sustained. However, careful inspection of the leaflet kinematics showed that one of the leaflets started to flutter slightly out-of-phase from the other leaflets. Therefore, the reduced fluctuations toward the end of the simulation were due to asynchronous leaflet motion and not due to reduced individual leaflet fluttering amplitudes. Further, we believe that there is sufficient time scale separation between central flow phenomena (e.g., fluttering at 40 Hz and the typical duration of the systolic phase approximately 0.3 s) and we found that the flow had enough time to establish the turbulent flow field during the early systolic phase. Therefore, we believe that the studied flow field was representative for the flow during peak systole. Additionally, the quasi-steady configuration had the advantage that turbulence statistics could use time-averaged quantities and that there were no artifacts due to cycle-to-cycle variations which are known to occur in pulsatile flow past heart valves (Sotiropoulos et al., 2016).

## 5. Conclusion

The present computational study characterized the turbulent systolic flow downstream of a bovine BHV. Similar to the study by Lee et al. (2020), we validated our numerical model against experimental data obtained for the same valve design.

We identified a peak of turbulent dissipation approximately one diameter downstream of the valve orifice. This was also the region where the vortex rings broke down, that were shed at 40 Hz from the fluttering leaflet tips. The total turbulent dissipation was found to be accountable for 26% of the total pressure loss over the valve (TVPG) indicating that turbulence is a significant and detrimental factor for hemodynamic valve performance. Further, we found elevated turbulent viscous shear stresses up to 14 Pa which may be connected to shear-induced thrombocyte activation. This could indicate that BHV thrombogenicity (Chakravarty et al., 2017) is connected to the turbulent flow past the valve.

To our best knowledge, this computational study is the first to present turbulent spectra behind BHV. As pointed out by Quinlan and Dooley (2007), whose study was limited to data for mechanical heart valves by Yoganathan et al. (1986) and Liu et al. (2000), such velocity spectra are an important basis to study shear-induced thrombocyte activation. Finally, the present computational model allowed us to study for the first time the wall shear stress patterns along the AAo wall (Figure 14 and *WSS video* in the Supplementary Material). We found elevated levels of turbulent WSS which suggest a possible connection between BHV turbulence, endothelial lesions and long-term AAo health.

## Data Availability Statement

The raw data supporting the conclusions of this article will be made available by the authors, without undue reservation.

## Author Contributions

BB contributed to the development of computational model, running the computational model, post-processing, analysis and interpretation of data, writing and critical revision of the manuscript, and study design. LP contributed to the post-processing and analysis of experimental data and critical revision of the manuscript. DO contributed to the post-processing, analysis and interpretation of data, writing and critical revision of the manuscript, and study design and supervision. All authors contributed to the article and approved the submitted version.

## Conflict of Interest

The authors declare that the research was conducted in the absence of any commercial or financial relationships that could be construed as a potential conflict of interest.

## References

[B1] Alemu Y. Girdhar G. Xenos M. Sheriff J. Jesty J. Einav S.. (2010). Design optimization of a mechanical heart valve for reducing valve thrombogenicity—a case study with ats valve. ASAIO J. 56, 389–396. 10.1097/MAT.0b013e3181e65bf9 20613492

[B2] Aljalloud A. Shoaib M. Egron S. Arias J. Tewarie L. Schnoering H.. (2018). The flutter-by effect: a comprehensive study of the fluttering cusps of the perceval heart valve prosthesis. Interact. Cardiovasc. Thorac. Surg. 27, 664–670. 10.1093/icvts/ivy162 29788476

[B3] Armani D. Liu C. Aluru N. (1999). Re-configurable fluid circuits by pdms elastomer micromachining, in Technical Digest. IEEE International MEMS 99 Conference. Twelfth IEEE International Conference on Micro Electro Mechanical Systems (Cat. No. 99CH36291) (Orlando, FL: IEEE), 222–227.

[B4] Auricchio F. Conti M. Ferrara A. Morganti S. Reali A. (2014). Patient-specific simulation of a stentless aortic valve implant: the impact of fibres on leaflet performance. Comput. Methods Biomech. Biomed. Eng. 17, 277–285. 10.1080/10255842.2012.681645 22553900

[B5] Betts J. G Young K. A. Wise J. A. Johnson E. Poe B. Kruse D. H. (2013). Anatomy and Physiology. Houston, TX: OpenStax Available online at: https://openstax.org/books/anatomy-and-physiology/pages/1-introduction

[B6] Chakravarty T. Søndergaard L. Friedman J. De Backer O. Berman D. Kofoed K. F.. (2017). Subclinical leaflet thrombosis in surgical and transcatheter bioprosthetic aortic valves: an observational study. Lancet 389, 2383–2392. 10.1016/S0140-6736(17)30757-2 28330690

[B7] Davies P. F. Remuzzi A. Gordon E. J. Dewey C. F. Gimbrone M. A. (1986). Turbulent fluid shear stress induces vascular endothelial cell turnover *in vitro*. Proc. Natl. Acad. Sci. U.S.A. 83, 2114–2117. 10.1073/pnas.83.7.2114 3457378 PMC323241

[B8] Ge L. Dasi L. P. Sotiropoulos F. Yoganathan A. P. (2008). Characterization of hemodynamic forces induced by mechanical heart valves: reynolds vs. viscous stresses. Ann. Biomed. Eng. 36, 276–297. 10.1007/s10439-007-9411-x 18049902

[B9] Haj-Ali R. Marom G. Zekry S. B. Rosenfeld M. Raanani E. (2012). A general three-dimensional parametric geometry of the native aortic valve and root for biomechanical modeling. J. Biomech. 45, 2392–2397. 10.1016/j.jbiomech.2012.07.017 22854206

[B10] Hasler D. Landolt A. Obrist D. (2016). Tomographic PIV behind a prosthetic heart valve. Exp. Fluids 57:80 10.1007/s00348-016-2158-0

[B11] Hasler D. Obrist D. (2018). Three-dimensional flow structures past a bio-prosthetic valve in an *in-vitro* model of the aortic root. PLoS ONE 13:e0194384. 10.1371/journal.pone.0194384 29547668 PMC5856406

[B12] Hatoum H. Dollery J. Lilly S. M. Crestanello J. Dasi L. P. (2019). Impact of patient-specific morphologies on sinus flow stasis in transcatheter aortic valve replacement: an *in vitro* study. J. Thorac. Cardiovasc. Surg. 157, 540–549. 10.1016/j.jtcvs.2018.05.086 29980299 PMC6056004

[B13] Hedayat M. Asgharzadeh H. Borazjani I. (2017). Platelet activation of mechanical versus bioprosthetic heart valves during systole. J. Biomech. 56, 111–116. 10.1016/j.jbiomech.2017.03.002 28347474

[B14] Hedayat M. Borazjani I. (2019). Comparison of platelet activation through hinge vs bulk flow in bileaflet mechanical heart valves. J. Biomech. 83, 280–290. 10.1016/j.jbiomech.2018.12.003 30579576

[B15] Holzapfel G. A. Gasser T. C. Ogden R. W. (2000). A new constitutive framework for arterial wall mechanics and a comparative study of material models. J. Elastic. Phys. Sci. Solids 61, 1–48. 10.1007/0-306-48389-0_1

[B16] Jahren S. E. Heinisch P. P. Hasler D. Winkler B. M. Stortecky S. Pilgrim T.. (2018). Can bioprosthetic valve thrombosis be promoted by aortic root morphology? An *in vitro* study. Interact. Cardiovasc. Thorac. Surg, Milano. 27, 108–115. 10.1093/icvts/ivy039 29481667

[B17] Jahren S. E. Heinisch P. P. Wirz J. Winkler B. Carrel T. Obrist D. (2015). Hemodynamic performance of Edwards Intuity valve in a compliant aortic root model, in 2015 37th Annual International Conference of the IEEE Engineering in Medicine and Biology Society (EMBC) (Milan: IEEE), 3315–3318. 10.1109/EMBC.2015.7319101 26737001

[B18] Jeong J. Hussain F. (1995). On the identification of a vortex. J. Fluid Mech. 285, 69–94. 10.1017/S0022112095000462

[B19] John M. O. Obrist D. Kleiser L. (2016). Secondary instability and subcritical transition of the leading-edge boundary layer. J. Fluid Mech. 792, 682–711. 10.1017/jfm.2016.117

[B20] Kamensky D. Hsu M.-C. Schillinger D. Evans J. A. Aggarwal A. Bazilevs Y.. (2015). An immersogeometric variational framework for fluid–structure interaction: Application to bioprosthetic heart valves. Comput. Methods Appl. Mech. Eng. 284, 1005–1053. 10.1016/j.cma.2014.10.040 25541566 PMC4274080

[B21] Kheradvar A. Groves E. M. Goergen C. J. Alavi S. H. Tranquillo R. Simmons C. A.. (2015). Emerging trends in heart valve engineering: part II. Novel and standard technologies for aortic valve replacement. Ann. Biomed. Eng. 43, 844–857. 10.1007/s10439-014-1191-5 25449148

[B22] Krause R. Zulian P. (2016). A parallel approach to the variational transfer of discrete fields between arbitrarily distributed unstructured finite element meshes. SIAM J. Sci. Comput. 38, C307–C333. 10.1137/15M1008361

[B23] Lee J. H. Rygg A. D. Kolahdouz E. M. Rossi S. Retta S. M. Duraiswamy N.. (2020). Fluid–structure interaction models of bioprosthetic heart valve dynamics in an experimental pulse duplicator. Ann. Biomed. Eng. 48, 1475–1490. 10.1007/s10439-020-02466-4 32034607 PMC7154025

[B24] Lee S.-W. Antiga L. Steinman D. A. (2009). Correlations among indicators of disturbed flow at the normal carotid bifurcation. J. Biomech. Eng. 131:061013. 10.1115/1.3127252 19449967

[B25] Lim W. Chew Y. Chew T. Low H. (1998). Steady flow dynamics of prosthetic aortic heart valves: a comparative evaluation with PIV techniques. J. Biomech. 31, 411–421. 10.1016/S0021-9290(98)00026-8 9727338

[B26] Liu J. Lu P. Chu S. (2000). Turbulence characteristics downstream of bileaflet aortic valve prostheses. J. Biomech. Eng. 122, 118–124. 10.1115/1.429643 10834151

[B27] Min Yun B. Aidun C. K. Yoganathan A. P. (2014). Blood damage through a bileaflet mechanical heart valve: a quantitative computational study using a multiscale suspension flow solver. J. Biomech. Eng. 136:101009. 10.1115/1.4028105 25070372 PMC4151159

[B28] Moore B. Dasi L. P. (2014). Spatiotemporal complexity of the aortic sinus vortex. Exp. Fluids 55:1770. 10.1007/s00348-014-1770-0 25067881 PMC4106046

[B29] Morbiducci U. Ponzini R. Nobili M. Massai D. Montevecchi F. M. Bluestein D.. (2009). Blood damage safety of prosthetic heart valves. shear-induced platelet activation and local flow dynamics: a fluid–structure interaction approach. J. Biomech. 42, 1952–1960 10.1016/j.jbiomech.2009.05.014 19524927

[B30] Nestola M. G. C. Becsek B. Zolfaghari H. Zuian P. De Marinis D. Krause R. (2019). An immersed boundary method for fluid-structure interaction based on variational transfer. J. Comput. Phys 398:108884 10.1016/j.jcp.2019.108884

[B31] Obrist D. Henniger R. Kleiser L. (2012). Subcritical spatial transition of swept Hiemenz flow. Int. J. Heat and Fluid Flow 35, 61–67. 10.1016/j.ijheatfluidflow.2012.01.012

[B32] Peacock J. A. (1990). An *in vitro* study of the onset of turbulence in the sinus of valsalva. Circ. Res. 67, 448–460. 10.1161/01.RES.67.2.448 2376081

[B33] Peskin C. S. (2002). The immersed boundary method. Acta Numer. 11, 479–517. 10.1017/S0962492902000077

[B34] Pope S. (2000). Turbulent Flows. Cambridge: Cambridge University Press.

[B35] Quinlan N. J. Dooley P. N. (2007). Models of flow-induced loading on blood cells in laminar and turbulent flow, with application to cardiovascular device flow. Ann. Biomed. Eng. 35, 1347–1356. 10.1007/s10439-007-9308-8 17458700

[B36] Reul H. Vahlbruch A. Giersiepen M. Schmitz-Rode T. Hirtz V. Effert S. (1990). The geometry of the aortic root in health, at valve disease and after valve replacement. J. Biomech. 23, 181–191. 10.1016/0021-9290(90)90351-3 2312522

[B37] Reynolds W. Hussain A. (1972). The mechanics of an organized wave in turbulent shear flow. part 3. theoretical models and comparisons with experiments. J. Fluid Mech. 54, 263–288. 10.1017/S0022112072000679

[B38] Sotiropoulos F. Le T. B. Gilmanov A. (2016). Fluid mechanics of heart valves and their replacements. Annu. Rev. Fluid Mech. 48, 259–283. 10.1146/annurev-fluid-122414-034314

[B39] Vennemann B. Rösgen T. Heinisch P. P. Obrist D. (2018). Leaflet kinematics of mechanical and bioprosthetic aortic valve prostheses. ASAIO J. 64, 651–661. 10.1097/MAT.0000000000000687 29045279

[B40] Yoganathan A. P. He Z. Casey Jones S. (2004). Fluid mechanics of heart valves. Annu. Rev. Biomed. Eng. 6, 331–362. 10.1146/annurev.bioeng.6.040803.140111 15255773

[B41] Yoganathan A. P. Woo Y.-R. Sung H.-W. (1986). Turbulent shear stress measurements in the vicinity of aortic heart valve prostheses. J. Biomech. 19, 433–442. 10.1016/0021-9290(86)90020-5 2943742

