# Supplementary material for: Turbulent Systolic Flow Downstream of a Bioprosthetic Aortic Valve: Velocity Spectra, Wall Shear Stresses, and Turbulent Dissipation Rates
Source: Front Physiol. 2020 Sep 29;11:577188. doi: 10.3389/fphys.2020.577188 (PMC7550765; doi:10.3389/fphys.2020.577188)
Supplement: Supplementary file 1 [file Data_Sheet_1.PDF]

# Supplementary Material

## 1 EXPERIMENTAL DATA FROM TOMOGRAPHIC PIV FOR COMPARISON

For validation of the computational model, we present here additional results extracted from the experimental study by Hasler and Obrist (2018). Besides inherent uncertainties in material parameters for the valve tissue, the experimental setup differed from the computational model in three main aspects: First, the experiment used a slightly tapered geometry for the AAo leading to an acceleration of the bulk flow in the AAo which is not present in the computational model. Second, the inflow was probably more disturbed in the experiment than in the computational model, because of the design of the left ventricle in the experimental flow loop. Third, the pulsatile character of the experimental configuration called for a phase-averaging over multiple pulses to obtain Reynolds decomposed flow fields with mean flow and fluctuations. The phase averaging of a scalar quantity $q(t)$ is defined as

$$\langle q \rangle_t = \frac{1}{N_P} \sum_{j=1}^{N_P} q(t + jT), \tag{S1}$$

where $T = 60/72$ s is the period of the pulse and the averaging was done over $N_p = 16$ pulses.

Figure S1 shows rms values $\mathbf{v}'_{f,\mathrm{rms}}$ for the velocity fluctuations in the experiment. Note that these fluctuation values are not strictly turbulent fluctuations, because they may also include cycle-to-cycle variations (Sotiropoulos et al., 2016). Figure S2 shows the corresponding Reynolds stress values $\mathrm{RSS}_{\mathrm{max}}$. Finally, figure S3 shows velocity spectra $E_{33}$ (Pope, 2000, p. 225) for the experimental measurements and for the compuational results. The data was extracted from the flow fields on the centerline at a height of $z = 0.03$ and $0.05\,\mathrm{m}$.

## REFERENCES


Hasler, D. and Obrist, D. (2018). Three-dimensional flow structures past a bio-prosthetic valve in an in-vitro model of the aortic root. *PLoS one* 13, 1–22

Pope, S. (2000). *Turbulent Flows* (Cambridge University Press)

Sotiropoulos, F., Le, T. B., and Gilmanov, A. (2016). Fluid mechanics of heart valves and their replacements. *Annual Review of Fluid Mechanics* 48, 259–283

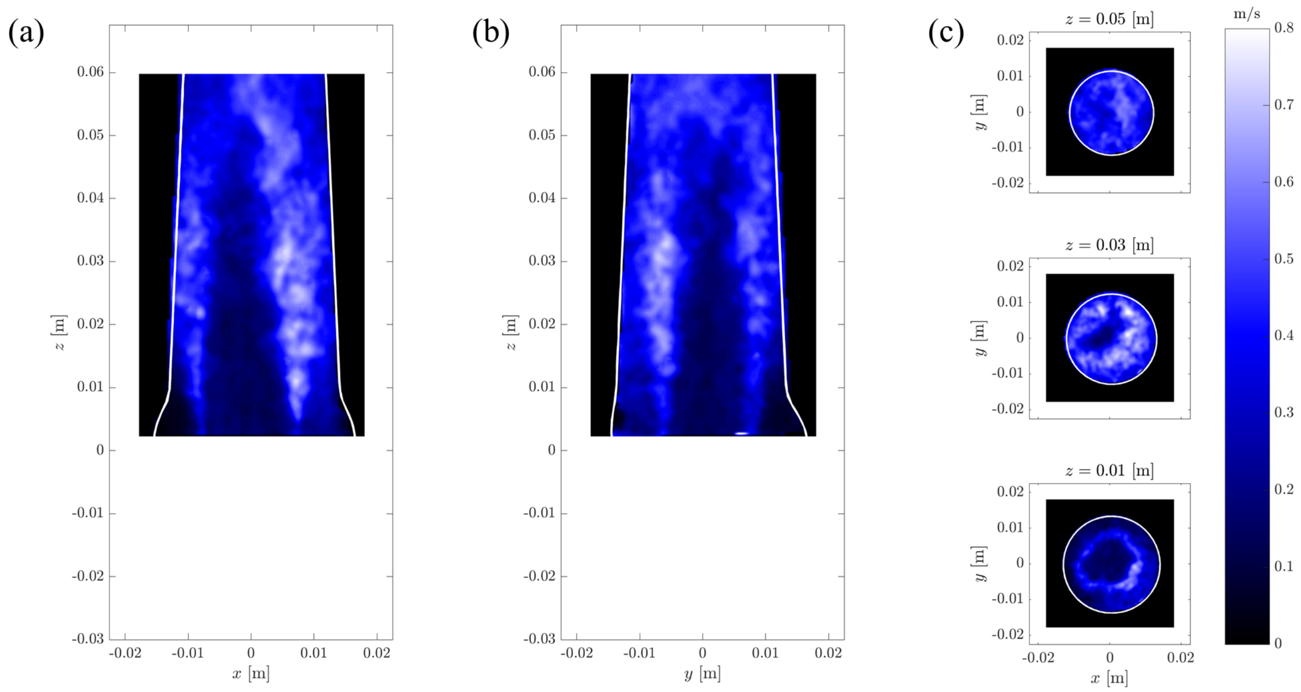


**Figure S1.** Root-mean-square fluctuation velocity values $\mathbf{v}'_{f,\mathrm{rms}}$ in different cross-sections of the PIV measurement domain (same cross-sections as in Figure 7).

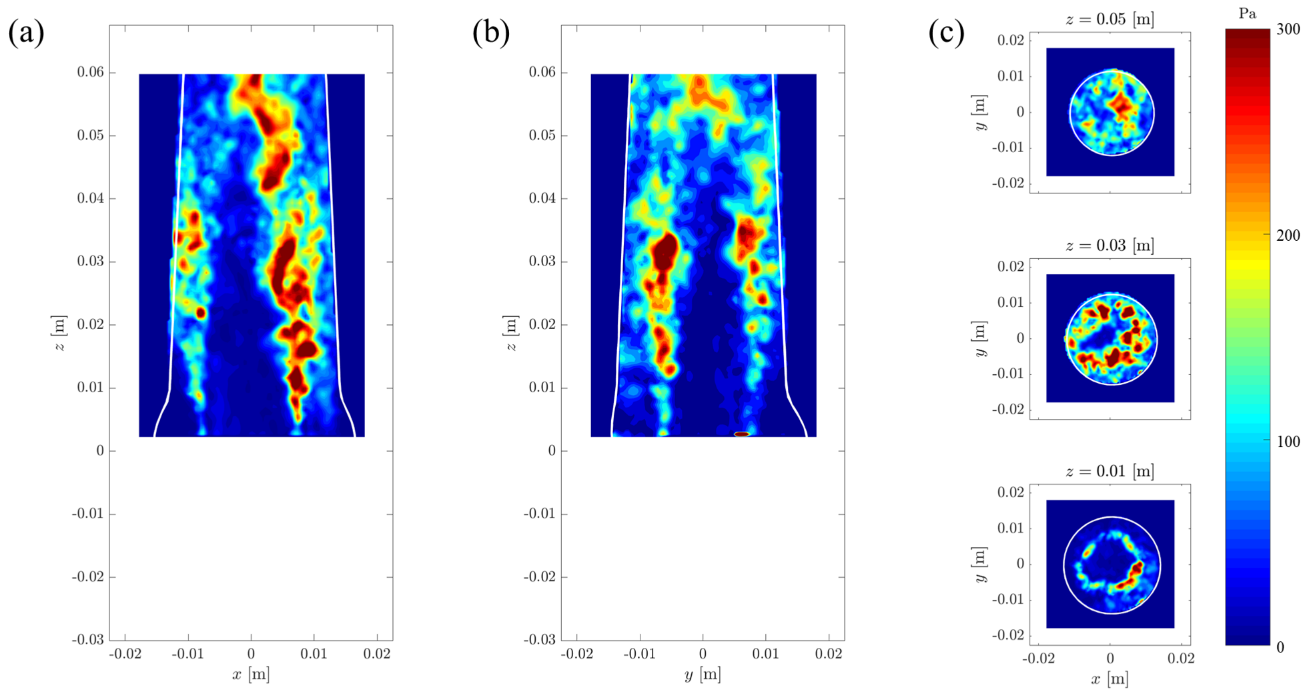


**Figure S2.** Maximum Reynolds shear stresses $\mathrm{RSS}_{\mathrm{max}}$ in different cross-sections of the PIV measurement domain (same cross-sections as in Figure 7).

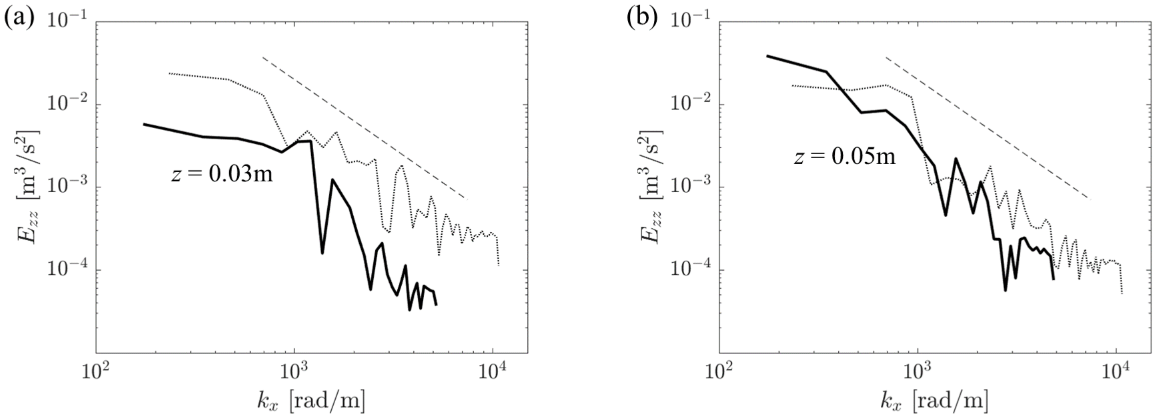


**Figure S3.** Velocity spectra $E_{33}(k_x)$ at different locations in the flow field: (a) $(x, y, z) = (0, 0, 0.03)\,\mathrm{m}$, (b) $(x, y, z) = (0, 0, 0.05)\,\mathrm{m}$. The dashed line shows a $-5/3$ decay rate typical for a turbulent inertial subrange. The dotted lines show the spectra obtained from computational data (same as in Figure 13) for comparison.
